# Underlying mechanisms of ketotherapy in heart failure: current evidence for clinical implementations

**DOI:** 10.3389/fphar.2024.1463381

**Published:** 2024-10-24

**Authors:** Kun Liu, Yang Yang, Jing-Hua Yang

**Affiliations:** Clinical Systems Biology Laboratories, The First Affiliated Hospital, Academy of Medical Sciences, Zhengzhou University, Zhengzhou, China

**Keywords:** heart failure, ketone bodies, cardiovascular protection, ketogenic treatment, energy metabolism, mechanisms of ketotherapy

## Abstract

Heart failure (HF) is a life-threatening cardiac syndrome characterized by high morbidity and mortality, but current anti-heart failure therapies have limited efficacy, necessitating the urgent development of new treatment drugs. Exogenous ketone supplementation helps prevent heart failure development in HF models, but therapeutic ketosis in failing hearts has not been systematically elucidated, limiting the use of ketones to treat HF. Here, we summarize current evidence supporting ketotherapy in HF, emphasizing ketone metabolism in the failing heart, metabolic and non-metabolic therapeutic effects, and mechanisms of ketotherapy in HF, involving the dynamics within the mitochondria. We also discuss clinical strategies for therapeutic ketosis, aiming to deepen the understanding of the characteristics of ketone metabolism, including mitochondrial involvement, and its clinical therapeutic potential in HF.

## 1 Introduction

Heart failure is an advanced stage of various cardiovascular diseases characterized by abnormal cardiac structure and function, resulting in reduced cardiac output and increased intra-cardiac pressure (Writing Committee and Members, 2022; Mascolo et al., 2022). Currently, the main approach for treating heart failure is to improve hemodynamics by regulating the renin-angiotensin-aldosterone system (RAAS) and the sympathetic nervous system (SNS), which includes positive inotropy, diuresis, and vasodilation (Mullens et al., 2017; Wu and Vaseghi, 2020; Tang and Kiang, 2020). Although these medications can alleviate clinical symptoms in the short term, they cannot alter the disease progression of heart failure, ultimately resulting in the persistence of high readmission rates and mortality among patients (Ponikowski et al., 2016). Therefore, identifying new therapeutic targets is a critical issue that needs to be addressed. Among them, ketone body metabolism has become a hot therapeutic target for cardiopharmacology.

**FIGURE 1:**
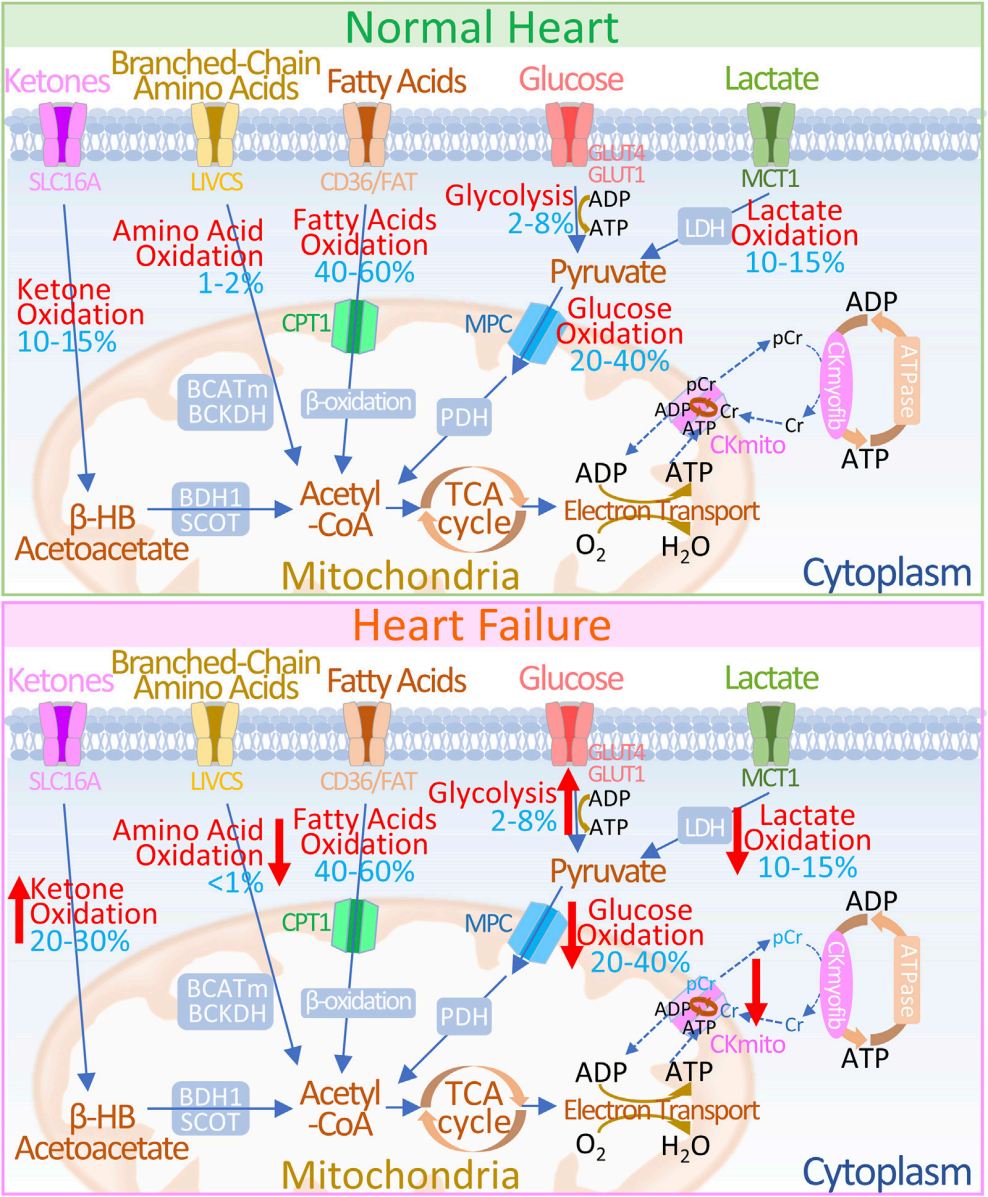
Overview of Energy Metabolism in Normal and Failing Hearts **(UP)** In normal hearts: 1) Glucose is transported into cells via GLUT1 or GLUT4 and undergoes glycolysis to produce pyruvate. 2) Lactate, taken up by MCT, is converted to pyruvate through LDH. 3) Pyruvate is transported to the mitochondria through the MPC and is converted to acetyl-CoA via PDH. 4) Fatty acids are transported into cells by CD36/FATP-1, esterified to fatty acyl-CoA, and then transferred to the mitochondria via CPT-1 and CPT-2 for β-oxidation. 5) Ketones (β-HB) are transported by SLC16A1 and oxidized by BDH1 to acetoacetate (AcAc), which is further activated to AcAc-CoA by SCOT, producing acetyl-CoA. 6) Branched-chain amino acids (BCAAs) are transported via LIVCS and converted to ketoacids in the mitochondria by BCATm. Acetyl-CoA and succinyl-CoA are subsequently formed by BCKDH intermediates.7) Acetyl-CoA generated from fatty acid β-oxidation, glucose oxidation, ketone oxidation, and BCAA oxidation enters the TCA cycle, producing FADH2 and NADH, which then enter the electron transport chain, consuming O2 to generate ATP. 8) The creatine kinase reaction rapidly and reversibly converts phosphocreatine (PCr) and ADP to ATP and creatine (Cr), which serve as the major energy reserve of the heart. **(DOWN)** In failing hearts, the following changes occur in energy metabolism pathways: 1) Increased ketone oxidation: Ketones are utilized more efficiently for energy production. 2) Altered amino acid oxidation: BCAA metabolism may be upregulated to compensate for reduced glucose availability. 3) Reduced fat oxidation: Fatty acid oxidation may be impaired due to decreased fatty acid transport or utilization. 4) Altered glycolysis and lactate metabolism: There might be a shift towards increased glycolysis and lactate production to meet energy demands. 5) Decreased glucose oxidation: Glucose utilization may be reduced due to insulin resistance or impaired glucose transport. 6) Changes in ATP production: The relative contribution of each pathway to ATP generation may be altered. Upward red arrows indicate increases, and downward red arrows indicate decreases. 7) Alteration in energy transfer: In HF, abnormalities in creatine kinase metabolism manifest as decreased levels of CK reactants, alterations in CK isoform, and diminished overall CK activity. The blue numbers indicate the contribution of each pathway to overall ATP production. Abbreviations: GLUT1/4: Glucose Transporter 1 and 4, MPC: Mitochondrial Pyruvate Carrier, PDH: Pyruvate Dehydrogenase, MCT: Monocarboxylate Transporter, LDH: Lactate Dehydrogenase, CD36/FAT: CD36/Fatty Acid Transporter, CPT-1: Carnitine Palmitoyl Transferase 1, FADH2: Flavin Adenine Dinucleotide, NADH: Nicotinamide Adenine Dinucleotide, ATP: Adenosine Triphosphate, ADP: Adenosine Diphosphate, TCA: Tricarboxylic Acid Cycle, βOHβ: β-hydroxybutyrate, SLC16A1: Solute Carrier Family 16 Member 1, BDH1: β-Hydroxybutyrate Dehydrogenase 1, SCOT: Succinyl-CoA: 3-Oxoacid Coenzyme A Transferase, LIVCS: L-type Amino Acid Transporter Vesicular Carrier System, BCATm: Mitochondrial Branched-Chain Amino Acid Transaminase, BCKDH: Branched-Chain α-Ketoacid Dehydrogenase Complex, Ckmyofib: Myofibrillar Creatine Kinase, CKmito: Mitochondrial Creatine Kinase, Cr: Creatine, pCr: Phosphocreatine.

The maintenance of cardiac function depends on the continuous generation and efficient utilization of adenosine triphosphate (ATP) (Doenst et al., 2013). With metabolic flexibility, the heart can adjust the proportions of different metabolic substrates according to energy requirements, substrate availability, and the body’s nutritional status (Lopaschuk and Ussher, 2016; Lopaschuk et al., 2021). Under physiological conditions, the ATP required for myocardial physiological function mainly comes from the metabolism of fatty acids (FAs), glucose, ketone bodies, lactic acid, amino acids and other substances (Shi and Qiu, 2022). Mitochondrial oxidation of fatty acids and glucose is the main source of energy required for the adult heart, providing more than 90% of ATP (De Jong and Lopaschuk, 2017), while the utilization of energy substrates such as ketones, lactate, and amino acids is minimal (Ussher et al., 2016; Lopaschuk et al., 2010). However, in heart failure, pathological cardiac structure and functional reconstruction are often accompanied by significant reshaping in energy generation and transfer reshaping, manifested as a significant decrease in the metabolic efficiency of fatty acids and carbohydrates for energy but an increase in ketone oxidation (Lopaschuk and Ussher, 2016; Shi and Qiu, 2022; Sun et al., 2016; Gupta et al., 2012; Keceli et al., 2022) (Figure 1).

Changes in ketone metabolism during heart failure are complex and depend on the severity and type of heart failure and on comorbidities such as obesity and type 2 diabetes (Lopaschuk et al., 2021). Importantly, alterations in the cardiac metabolism of ketone bodies contribute to the severity of heart failure. The pharmacological targeting of ketone body metabolism has emerged as a novel therapeutic approach to improve cardiac efficiency, reduce energy deficiency, and enhance cardiac function in failing hearts (Braunwald, 2013). However, whether increased ketone metabolism in heart failure patients is adaptive remains unclear. In addition, the functional regulation and underlying mechanisms of ketone bodies in failing hearts, including metabolic and non-metabolic effects, have not been systematically elucidated.

Here, we review the therapeutic effects of ketones in heart failure, emphasizing the metabolic alteration of ketone bodies in pathological restructuring, the metabolic and non-metabolic functions and mechanisms of ketone metabolism in pathological cardiac remodeling, and clinical strategies for therapeutic ketosis, which are highly important for understanding the underlying mechanisms and evidence of therapeutic ketosis for pathological cardiac remodeling.

## 2 Ketone metabolism in normal and failing heart

### 2.1 Production and energetic properties of ketone bodies

**FIGURE 2:**
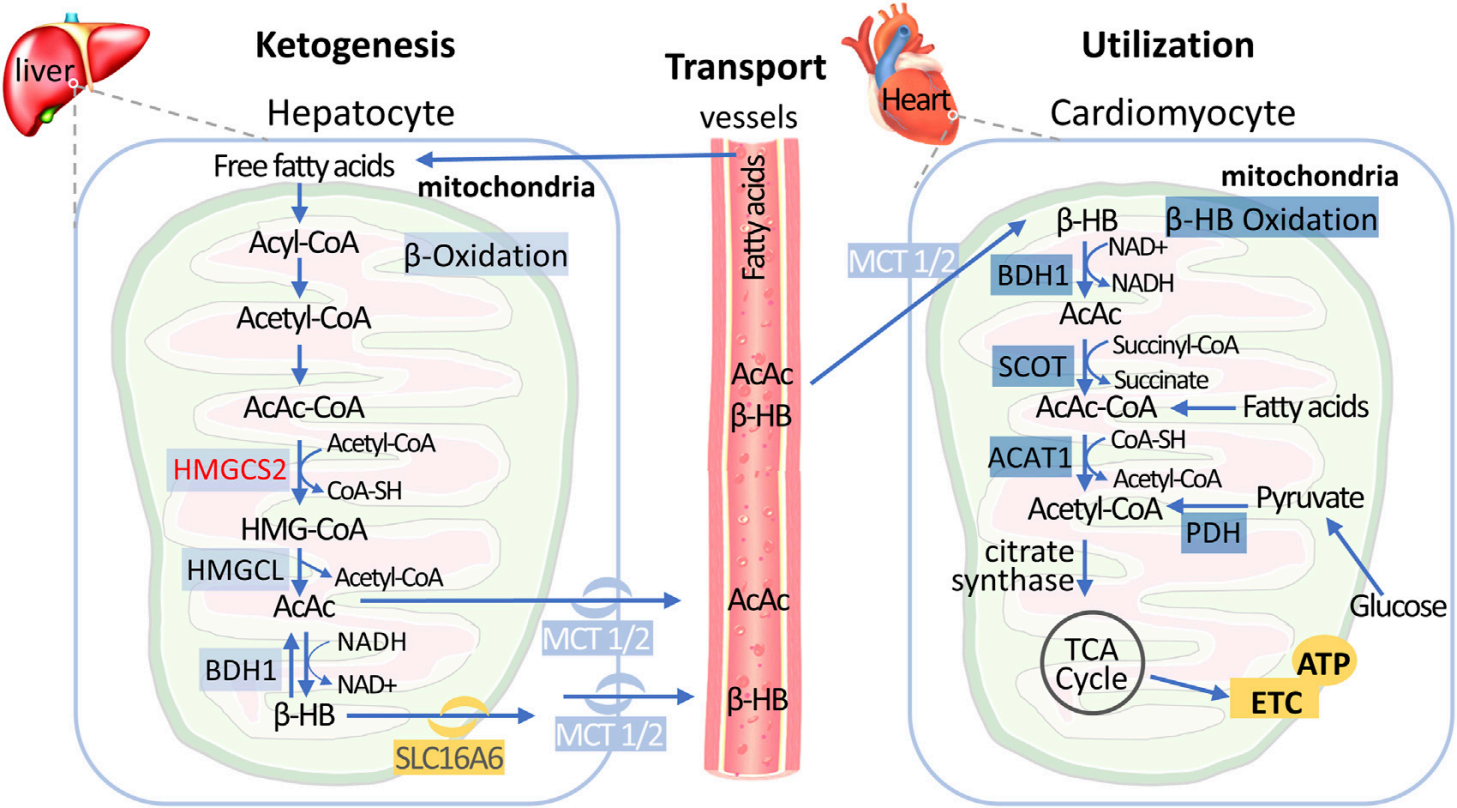
Metabolic Pathways of Hepatic Ketogenesis and Myocardial Ketone Utilization Free fatty acids transported from circulation undergo fatty acid oxidation in the liver mitochondria to produce acetyl-CoA. Subsequently, ketogenesis process utilizes acetyl-CoA to ultimately generate two mature ketone bodies: acetoacetate (AcAc) and β-hydroxybutyrate (β-HB). After released into the circulation and import it into cardiomyocytes by the monocarboxylate transporter (MCT), AcAc and β-HB are converted back to acetyl-CoA via cardiomyocyte mitochondrial ketolysis, which can be subsequently metabolized in the tricarboxylic acid cycle to generate ATP. Of note, acetyl-CoA is also produced by fatty acids and glycolytic pathways in the early stages of failing heart, but decreased in end-stage HF. Abbreviations: β-HB, β-hydroxybutyrate; HMGCS2, 3-hydroxy-3-methyl-glutaryl-CoA synthase; HMGCL, 3-hydroxy-3-methyl-glutaryl-CoA lyase; BDH, BHB dehydrogenase; MCT, monocarboxylate transporter; SCOT, succinyl-CoA: 3-oxoacid CoA transferase; ACAT, acetyl-CoA acetyltransferase; PDH, pyruvate dehydrogenase; TCA, tricarboxylic acid.

Biologically, ketone bodies serve as important energy reserves to maintain metabolic homeostasis in the body during stress and starvation, and ketone body levels are typically low in non-fasting conditions (Puchalska and Crawford, 2017). Endogenously produced ketone bodies include β-hydroxybutyrate (β-HB), acetoacetate (AcAc), and acetone, which are primarily derived from the conversion of fatty acids in the liver (Figure 2) (Zhang et al., 2011). During fasting, hepatocytes transport mobilized fatty acids into the mitochondria, where they undergo β-oxidation to generate multiple acetyl-CoA molecules. Acetyl-CoA can be further converted to acetoacetate through various enzymatic reactions, and acetoacetate can also be reduced back to β-HB for further utilization (Sun et al., 2016). The production of endogenous ketone bodies is influenced by hormonal signals, transcriptional regulation, and posttranslational modifications (Puchalska and Crawford, 2017). Ketone bodies can be cleared by the kidneys or lungs, converted into lipids, or transported to extrahepatic tissues for utilization.

Ketone bodies are increasingly recognized as important energy substrates for the heart, with a better balance between the quantity and efficiency of energy production (De Jong and Lopaschuk, 2017; Lopaschuk, 2017; Selvaraj et al., 2020; Qian and Wang, 2020). Considering the oxygen consumption of ATP production, ketone body oxidation is considered to be a relatively efficient oxidation pathway because the phosphate-to-oxygen (P/O) ratio of ketone body oxidation is 2.5, which is more favorable than fatty acid oxidation (P/O ratio of 2.3). In addition, by calculating the ATP produced per 2 carbon molecules, the oxidation reaction of ketone bodies was shown to produce more ATP than glucose. In fact, adding ketone bodies to the glucose infusion significantly increased cardiac output and efficiency in an isolated rat heart model, similar to insulin addition (Keon et al., 1995).

### 2.2 Ketones metabolism in heart

The myocardium is a tissue with high enzyme activity for ketone metabolism, and ketone bodies are easily metabolized by the heart. The uptake and utilization of ketone bodies by myocardial cells are the result of the synergistic action of multiple enzymes (Figure 2) (Puchalska and Crawford, 2017; Janardhan et al., 2011): 1) Myocardial cells take up circulating ketone bodies through monocarboxylic acid transporters and oxidize them to acetoacetate by β-hydroxybutyric acid dehydrogenase 1 (BDH1) in mitochondria; 2) acetoacetate is further activated to acetoacetyl-CoA by 3-ketozcid CoA transferase 1 (SCOT1); and 3) acetyl-CoA acetyltransferase (ACAT) catalyzes the thiolysis reaction, generating two molecules of acetyl-CoA that enter the tricarboxylic acid cycle. Studies have shown that SCOT1 knockout mice exhibit more severe cardiac structural and functional impairments during heart failure (Aubert et al., 2016), indicating that impaired ketone metabolism exacerbates myocardial injury under cardiac stress conditions.

Under non-fasting conditions, the myocardium rarely takes up ketone bodies and primarily relies on fatty acid oxidation (FAO) and glucose oxidation for energy (Ho et al., 2019), but when circulating ketone bodies increase, the heart preferentially utilizes ketone bodies for energy while reducing FAO and glucose oxidation (Stanley et al., 2003; Ziegler et al., 2002). Among them, β-HB is the major ketone body oxidized in the heart.

### 2.3 Increased ketone metabolism in HFrEF

Dramatic changes in energy metabolism are evident throughout the entire process, from pathological myocardial remodeling to heart failure. During heart failure, mitochondrial oxidative capacity is reduced by increased reactive oxygen species (ROS), dysregulated mitochondrial Ca^2+^ homeostasis, impaired mitochondrial dynamics, mitochondrial autophagy and other adverse factors, leading to a reduced capacity and efficiency of fatty acid and glucose oxidation (De Jong and Lopaschuk, 2017). However, animal and human HF models have demonstrated the increased utilization of ketone bodies (Aubert et al., 2013), possibly because ketone oxidation bypasses the dysregulation of the β-oxidation pathway and pyruvate dehydrogenase complex.

Many studies support the concept that ketones are alternative metabolic substrates for failing hearts. During the progression of HFrEF induced by myocardial infarction in mice, not only was the expression of SLC16A1 increased to mediate the cardiac uptake of ketones but also the expression of ketone oxidation enzymes, such as BDH1, increased in cardiomyocytes (Aubert et al., 2016). By quantifying the utilization rate of metabolic substrates in arterial and venous blood samples, a study reported an approximately 100% increase in ketone oxidation in patients with heart failure with a reduced ejection fraction (HFrEF) (Funada et al., 2009). In addition, especially among patients with end-stage heart failure, fasting-induced increases in circulating ketone bodies are aggravated, and the content of the ketogenic derivative β-hydroxybutyryl-CoA (βHB-CoA) is also dramatically increased (Selvaraj et al., 2020). Overall, the specific mechanisms underlying the increase in ketone metabolism during HF progression are not yet clear but may be related to the following factors (Kolwicz et al., 2016): 1) cardiac disease may lead to metabolic remodeling of the body, increasing ketone synthesis in the liver; 2) pathological remodeling of the heart may enable myocardial cells to more effectively take up ketone bodies from the blood; and 3) certain metabolic pathway changes in myocardial cells may activate the ketone utilization enzyme system.

Further research indicated that enhanced ketone body metabolism is a compensatory change. Heart failure (HF) mice with heart-specific BDH1 knockout exhibit more severe ventricular remodeling and dysfunction. Conversely, in a pressure overload HF model, overexpression of BDH1 can alleviate cardiac remodeling and DNA damage (Uchihashi et al., 2017). Additionally, during the progression of HFrEF, myocardial ketone body oxidation and impaired fatty acid oxidation occur simultaneously. Heart-specific SCOT knockout mice (in which terminal oxidation of β-hydroxybutyrate is prevented) exhibit increased fatty acid oxidation (Schugar et al., 2014), revealing the interrelated characteristics of myocardial ketone oxidation and fatty acid oxidation.

### 2.4 Ketone metabolism in HFpEF

The above studies examined the changes in ketone metabolism in HFrEF patients, but there is no consensus on the changes in ketone metabolism associated with heart failure with preserved ejection fraction (HFpEF). The alterations in ketone oxidation in HFpEF patients remain unclear. A 3-Hit mouse model revealed the complexity of HFpEF in relation to myocardial ketone oxidation (Deng et al., 2021). Despite increased circulating blood ketone levels, myocardial ketone oxidation did not increase in this HFpEF model, but a reduction in mitochondrial dysfunction induced by proinflammatory cytokines was observed, which is contrary to the findings in HFrEF. It is currently unclear whether hyperketonemia in HFpEF patients is associated with increased oxidation of other metabolic substrates in the myocardium (Du et al., 2014; Lommi et al., 1996). Additionally, serum metabolomics revealed greater levels of AcAc and β-HB in HFpEF patients than in HFrEF patients, suggesting that HFrEF patients have increased ketone consumption and rely more on ketones as an energy source than HFpEF patients do (Zordoky et al., 2015). However, the role of ketones in the development of HFpEF remains to be clearly determined, and future work is needed to elucidate the role of ketone metabolism in the progression of HFpEF.

## 3 Therapeutic effects of ketones in heart failure

**FIGURE 3:**
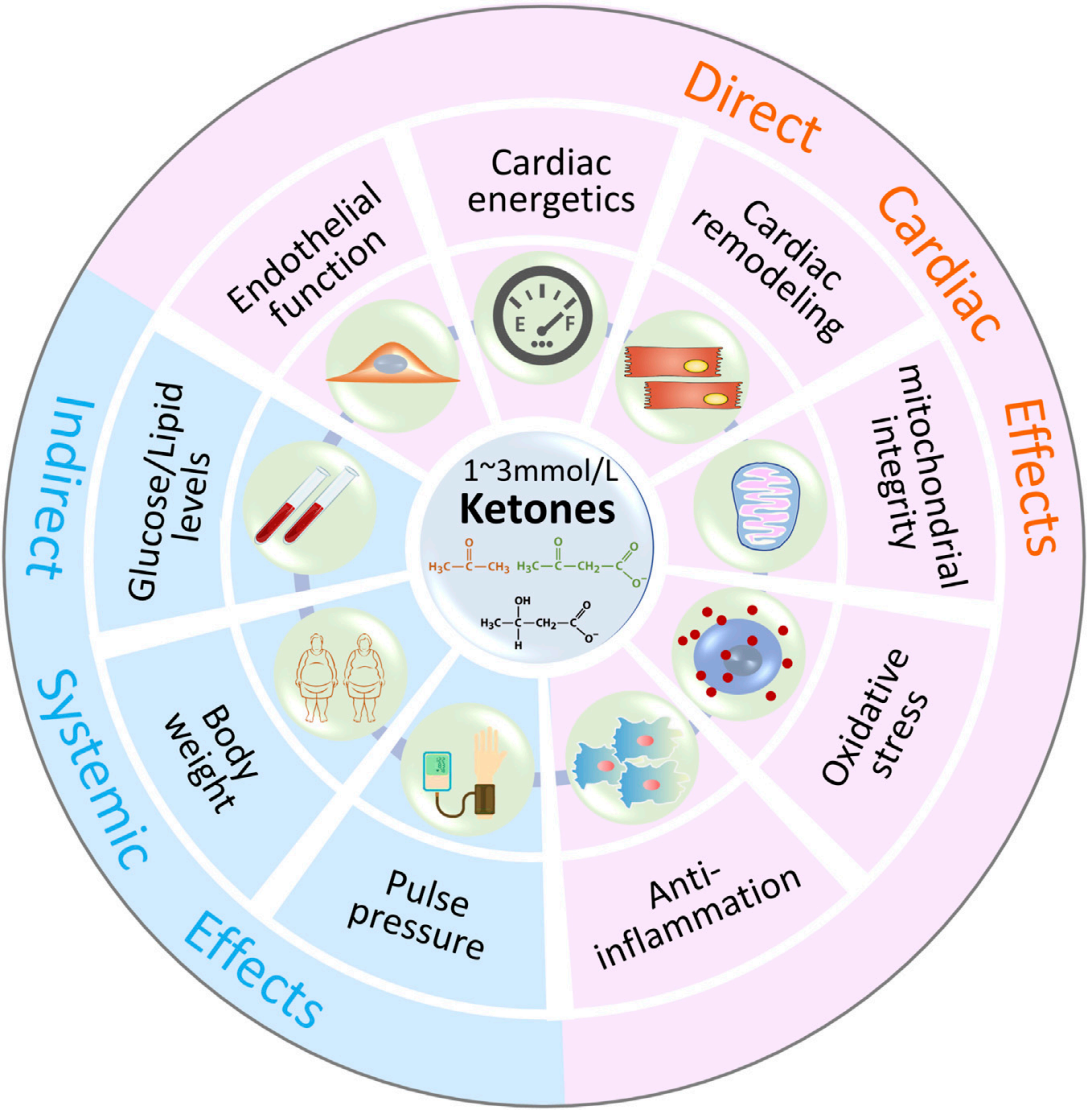
Metabolic and Non-Metabolic Effects of Ketone Bodies In addition to energy supplement as alternative substrates, ketone bodies (1–3 mmol/L β-HB) may have beneficial effects on oxidative stress, mitochondrial function, endothelial function, inflammation, cardiac remodeling and other indirect systemic effects, such as body weight, blood pressure, serum glucose and lipids levels.

Numerous studies support the protective effect of ketone bodies on the failing heart, and the cardioprotective effect of ketone bodies may not only be related to energy metabolism. As shown in Figure 3, ketones could have metabolic and non-metabolic beneficial effects on HF.

### 3.1 Energy supplement

The consumption of ketone bodies is approximately 3-fold greater in patients with heart failure than in healthy controls. In particular, when experiencing insufficient glucose supply (such as during fasting), approximately 16.4% of the heart’s ATP generation comes from ketone bodies in patients with HFrEF (Murashige et al., 2020; Abdul Kadir et al., 2020). Numerous studies have revealed that the cardiac uptake of β-HB and the content of β-HB-CoA are both significantly increased in patients with HFrEF, end-stage heart failure, or aortic stenosis (Bedi et al., 2016; Voros et al., 2018), indicating that ketone bodies are important alternative substrates for heart failure and that increased ketone metabolism is an adaptive response of the heart to inadequate energy supply.

### 3.2 Cardiac remodeling

Research suggests that insufficient ketone production may be associated with adverse ventricular remodeling, and ketone supplementation may help improve cardiac remodeling (Schugar et al., 2014; Yurista et al., 2021a), suggesting that ketone bodies may be involved in the myocardial response to pressure overload. Artificial aortic coarctation mice with cardiomyocyte-specific SCOT knockout exhibited disrupted myocardial mitochondrial and myofibril ultrastructures, increased left ventricular volume, and decreased ejection fraction (Schugar et al., 2014). Furthermore, several studies have revealed that ketone supplementation significantly improves the left ventricular ejection fraction in animals with myocardial infarction (MI) or heart failure and reduces ventricular mass, myocardial cell cross-sectional area, and atrial natriuretic peptide protein expression (Yurista et al., 2021a), which further demonstrates the protective role of ketone body metabolism on cardiac structure.

### 3.3 Mitochondrial integrity

Recently, studies have reported that ketone bodies play a role in maintaining mitochondrial integrity in heart disease (Huynh, 2016). Some research has shown that increasing ketone metabolism can alleviate the damage to the heart caused by myocardial ischemia‒reperfusion or myocardial infarction because ketone metabolism prevents a reduction in mitochondrial quantity and deterioration of mitochondrial function within cardiomyocytes (Al-Zaid et al., 2007; Snorek et al., 2012). Additionally, ketogenic diets can significantly extend the lifespan of mice with genetic lethal cardiomyopathy because they help maintain the integrity of cardiomyocytic mitochondrial structures and improve oxidative phosphorylation (Krebs et al., 2011). Research on heart-specific SCOT knockout mice with transverse aortic constriction (TAC) revealed disordered mitochondrial and myofibrillar microstructures in cardiomyocytes, accompanied by increased left ventricular volume and decreased ejection fraction (Schugar et al., 2014).

### 3.4 oxidative stress

Oxidative stress plays a crucial role in the cardiovascular system, participating in various signaling pathways contributing to pathological processes such as cardiac hypertrophy and myocardial cell apoptosis (Cinato et al., 2024; Liu et al., 2023). However, elevated levels of β-HB may serve as a compensatory response of the failing heart to oxidative stress (Nagao et al., 2016). In H9c2 cells, BDH1 overexpression reduces the generation of reactive oxygen species clusters, suggesting that increased utilization of ketone bodies may help decrease oxidative stress and improve cardiac remodeling (Uchihashi et al., 2017). In an oxidative stress cardiomyocyte model, β-HB may also induce the expression of the oxidative stress resistance gene FOXO3a and increase the expression of catalase, superoxide dismutase, and peroxiredoxin, thereby inhibiting the generation of reactive oxygen species clusters and reducing cell apoptosis (Nagao et al., 2016).

### 3.5 Anti-inflammation

Activation of the NOD-like receptor pyrin domain containing 3 (NLRP3) inflammasome in the heart leads to myocardial injury. However, in a heart failure mouse model, prolonged elevation of circulating ketone body levels alleviated NLRP3 inflammasome-mediated myocardial inflammation and improved cardiac function (Byrne et al., 2020; Youm et al., 2015). Elevated levels of β-hydroxybutyrate (β-HB) bind to the NLRP3 inflammasome, inhibiting its activity and thereby reducing the release of the inflammatory factors IL-1β and IL-18, slowing myocardial fibrosis and promoting the recovery of the ejection fraction during heart failure progression (Deng et al., 2021).

However, acetoacetic acid (AcAc) is considered to have pro-inflammatory effects. Elevated concentrations of AcAc, especially at high glucose levels, aggravate endothelial cell injury through oxidative stress and are associated with increased expression of TNF-α and MCP-1 in monocytes, accumulation of reactive oxygen species (ROS), and decreased cAMP levels (Jain et al., 2002). Therefore, the role of ketone bodies in anti-inflammatory mechanisms still needs further investigation.

### 3.6 Angiogenesis

In heart failure, angiogenesis is crucial for slowing myocardial injury and protecting the heart. Ketone oxidation can effectively prevent a decrease in vascular density in failing hearts, particularly by promoting the proliferation, migration, and sprouting angiogenesis of cardiac endothelial cells (Weis et al., 2022). In healthy mice, transient elevation of circulating ketones only induces proliferation of cardiac endothelial cells without affecting cardiac vascular density. However, in models of cardiac hypertrophy and heart failure, long-term elevation of ketone levels can further increase the proliferation of endothelial cells in mice (Weis et al., 2022).

## 4 Molecular signaling of Ketone therapy in heart failure

Lopaschuk and Ussher (Ho et al., 2019; Mayr et al., 2008) suggested that as an additional energy substrate, the amount of ATP produced by ketone oxidation does not exceed 20% of the total demand of the heart, which seems insufficient to meet the need for cardiac function (Figure 1). In other words, ketone bodies may exert their effects on the heart through mechanisms other than oxidative metabolism. Here, we review the metabolic and non-metabolic mechanisms of ketone bodies in resisting the pathological remodeling of failing hearts.

### 4.1 The oxidative metabolism of ketone bodies

Although the protective effects of ketone oxidation on the heart have been supported by several studies, the specific mechanisms involved have not been fully elucidated. According to current research, the protective effects of ketone oxidation in the heart may involve the following two processes. First, myocardial cells utilize energy produced by ketone oxidation more efficiently than fatty acid and glucose oxidation. Therefore, when the efficiency of fatty acid and glucose oxidation in myocardial cells decreases, ketones can serve as a “super fuel” to promptly replenish energy (Cotter et al., 2013). Second, ketone metabolism alleviates oxidative stress reactions, prevents excessive generation and accumulation of reactive oxygen species (ROS), inhibits lipid peroxidation reactions, and increases the antioxidant capacity of proteins, thereby improving mitochondrial function and enhancing ATP synthesis efficiency in cardiomyocytes (Puchalska and Crawford, 2017).

However, some studies have reached opposite conclusions. Wang et al. (2008) found that cardiac systolic function in mice with myocardial ischemia did not improve and may even be worsened by ketogenic diets. In another *in vitro* experiment where ketones were the sole energy source for cardiomyocytes, acute contractile dysfunction was observed. However, after supplementation with glucose, cardiac function significantly improved. This may be because ketones cannot provide important intermediates produced by glucose oxidation, which are crucial for the tricarboxylic acid cycle (Taegtmeyer, 2016). Additionally, Mayr et al. (2008) showed that increased ketone metabolism altered the excitability of myocardial cellular membranes, promoting the occurrence of arrhythmias. β-HB blocks transient K^+^ efflux in ventricular myocytes, resulting in prolonged action potential duration. Taken together, these findings indicate that the impact of ketone metabolism on cardiac function is not entirely consistent and may be regulated and influenced by multiple factors.

In conclusion, there is currently no consensus on the role and mechanisms of ketone oxidation metabolism in heart disease. This may be due to differences in disease states and the proportion of ketones in the energy substrate. Additionally, the above studies have some notable limitations, such as the lack of assessment of the relationship between increased ketone metabolism and ATP generation. Further research is needed to explore whether increased ketone oxidation metabolism in heart disease is accompanied by sufficient ATP production and to investigate the mechanisms of ketone metabolism in heart disease to better understand its potential therapeutic effects and safety.

### 4.2 Anti-oxidative stress

The protective effects of ketone bodies against oxidative stress injury not only result from their direct chemical targeting of reactive oxygen species (ROS) and free radicals but also from their regulation of gene expression to maintain redox homeostasis. In cardiomyocytes, ketone bodies can reduce oxidative stress-induced cell damage and apoptosis by attenuating ROS and enhancing antioxidant defense (Oka et al., 2021). β-HB can act as a direct antioxidant against hydroxyl radicals (Haces et al., 2008) and oxidize coenzyme Q (Veech et al., 2001; Kashiwaya et al., 2000), reducing the content of semiquinone in coenzyme Q. As important factors for resisting oxidative stress and electrophile attack, nuclear factor erythroid 2-related factor 2 (Nrf2) and other antioxidant defense target genes can be transcriptionally promoted by β-HB to prevent oxidative stress (Kolb et al., 2021).

However, several conflicting reports indicate that ketone bodies induce oxidative stress in various cardiovascular cells, including cardiomyocytes, smooth muscle cells, and endothelial cells (Pelletier and Coderre, 2007; Tian et al., 2014). However, some studies have shown the beneficial effects of ketone body-induced oxidative stress, as ketone bodies can initiate adaptive responses by activating major regulators of cytoprotection, including Nrf2, Sirt1/3, and AMPK(Kolb et al., 2021; Milder and Patel, 2012; Abdelmegeed et al., 2004).

### 4.3 Signaling transduction modulation

Recent research has shown that ketones can regulate cellular physiological and pathological processes by modulating signaling pathways (Newman and Verdin, 2014; Rojas-Morales et al., 2016). Among them, ketone bodies have been proven to be agonists of GPR81, GPR109A and GPR109B, which are defined as hydroxycarboxylic acid 1, 2 and 3 receptors (HCARs) and are G protein-coupled receptors (GPCRs), respectively.

One study identified β-HB as the only known endogenous ligand for GPR190A (Offermanns et al., 2011; Zhang et al., 2021a). When undergoing ketogenic diets, starvation, or ketoacidosis, the blood concentration of β-HB increases, which activates GPR190A to reduce lipolysis in adipose tissue, thereby decreasing the entry of nonesterified fatty acids into the liver for ketone synthesis and forming a negative feedback mechanism (Ahmed et al., 2009). Additionally, activation of GPR190A can enhance macrophage cholesterol efflux, reducing the formation of atherosclerotic plaques. In addition to being a GPR190A activator, β-HB has also been proven to antagonize GPR41, which can induce a decrease in intracellular cyclic adenosine monophosphate (cAMP), thereby reducing lipolysis. Furthermore, β-HB can also reduce sympathetic excitability and slow heart rate by activating GPR41, which has an impact on heart disease (Kimura et al., 2011).

In addition to the above targets, in various disease models, ketone bodies can also act on AMPK-FOXO3 (Forkhead box O3), FOXO1 (Bae et al., 2016), PPARγ coactivator 1 alpha (PGC-1α) (Kim et al., 2019), NADPH oxidase 4 (NOX4) (Kanikarla-Marie and Jain, 2015), Akt-mTOR (Li et al., 2017) and the MAPK signaling pathway (Li et al., 2022) to exert anti-inflammatory effects, endoplasmic reticulum stress and other pathophysiological processes, which are closely related to the occurrence and development of heart diseases.

### 4.4 Epigenetic and transcriptional regulation

**FIGURE 4:**
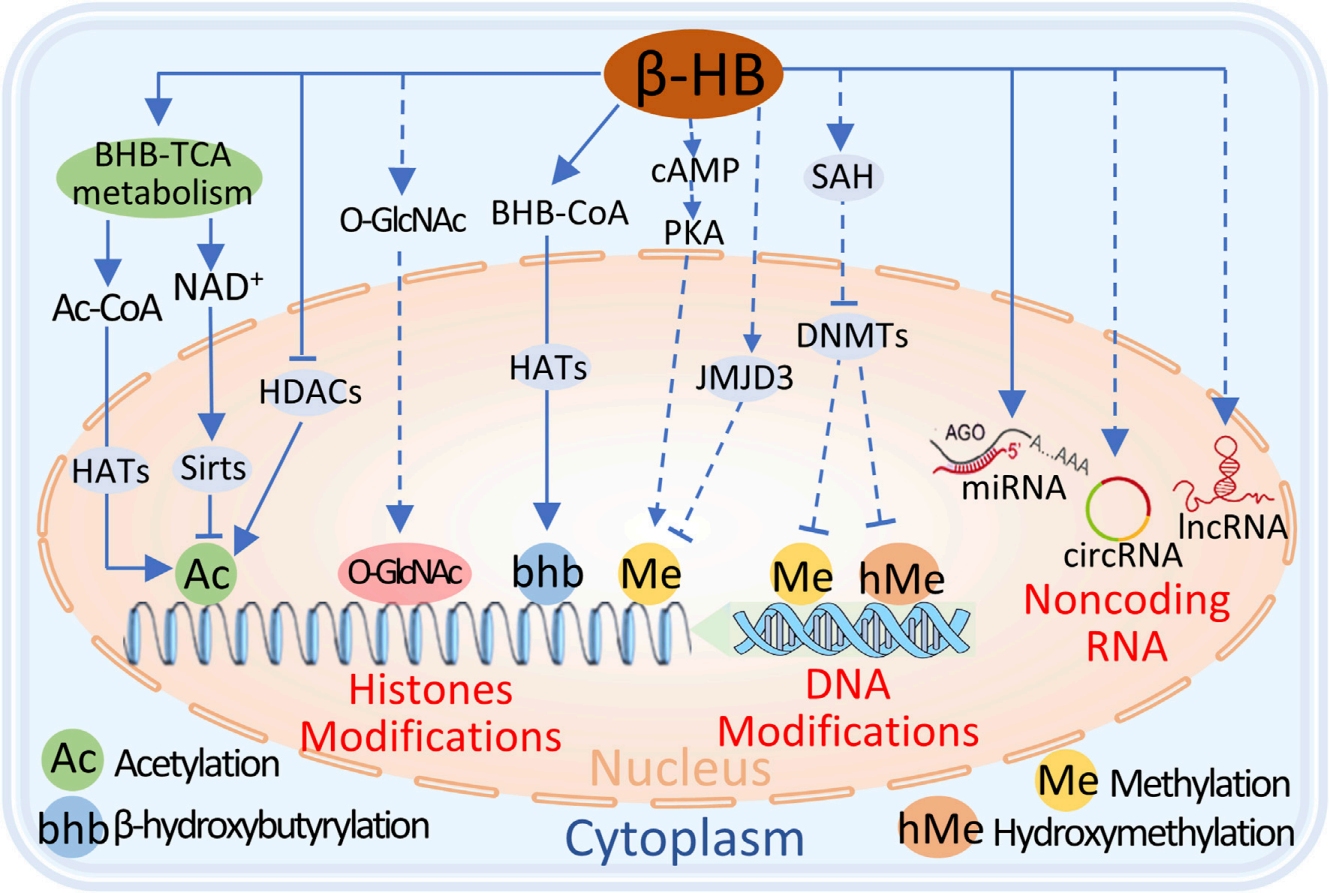
The potential mechanisms of BHB regulation of epigenetics include 1) BHB metabolism in mitochondria increases Ac-CoA, which acts as a substrate for histone acetyltransferases (HATs) to promote histone lysine acetylation. 2) Excessive NAD activates Sirts to promote histone lysine deacetylation. 3) BHB directly inhibits histone deacetylases (HDACs) to increase histone lysine acetylation. 4) BHB can be converted to BHB-CoA, and HATs promote histone lysine β-hydroxyisobutyrylation. 4) BHB promotes histone lysine methylation through cAMP/PKA signal, but indirectly inhibits histone lysine methylation through the upregulation of JMJD3. 5) BHB inhibits DNA methyltransferases (DNMTs) to block DNA cytosine methylation or hydroxymethylation. 6) Additionally, BHB regulates microRNA, circRNA, and lncRNA and affects gene expression. Dashed lines indicate the process requires further validation, and solid lines indicate the process has been proven.

Transcriptional reprogramming is one of the molecular mechanisms driving the pathophysiology of heart failure (HF), in which epigenetic events serve as molecular transducers of gene expression. Many reports have shown that ketones regulate gene expression in heart failure through epigenetic mechanisms, including posttranslational modifications of histones, DNA modifications, and posttranscriptional regulation of noncoding RNAs (He et al., 2023). (Figure 4)

#### 4.4.1 Posttranslational modifications of histones

Among the explored epigenetic factors, post-translational modifications of histones, especially histone acetylation and methylation, which can be modulated by ketone bodies, have attracted the most attention due to their potential as therapeutic targets for heart failure (Eom and Kook, 2014; Li et al., 2020a; Yang et al., 2020).

Shimazu et al. (2013) reported that β-HB can promote histone acetylation by inhibiting class I histone deacetylases (HDACs), thereby increasing the expression of antioxidative stress genes. Furthermore, acetyl-CoA generated from ketone oxidation is a potential factor leading to protein hyperacetylation and impacting organismal metabolism (Menzies et al., 2016). Recent studies have shown that in mice experiencing long-term fasting or diabetic ketoacidosis, β-HB can directly modify lysine residues in histone and nonhistone substrates through a newly discovered PTM called β-hydroxybutyrylation, thereby regulating organismal function (Xie et al., 2016; Liu et al., 2019). However, although relevant studies have demonstrated that ketone metabolism can regulate physiological processes through PTMs, there is currently no direct evidence of its impact on heart disease, and further research is needed in this area.

Indeed, recent evidence supports the notion that HDAC inhibitors, which were initially developed for various cancer treatments, can improve cardiac and pulmonary function in HFpEF animal models (Li et al., 2020a; McKinsey, 2012; Wallner et al., 2020). It is crucial to note that further research is needed to differentiate the effects of these inhibitors, as they could either directly target epigenetic regulation via histones or indirectly through non-histone targets, such as gene expression modulation and enzyme activity regulation (Jeong et al., 2018; Khan et al., 2018).

Recent studies have explored β-HB as a metabolic mediator of histone H3 methylation remodeling in the ischemic heart using human heart specimens and mouse and cardiomyocyte models (Gambardella et al., 2023). This study revealed that H3K27me2 and H3K36me1, which are specifically upregulated histone modifications in ischemic heart failure, are sensitive to β-HB levels. The increase in H3K27me2 and H3K36me1, as well as the mitochondrial dysfunction caused by the downregulation of the downstream transcription factor PGC1α, was significantly reduced by β-HB treatment, revealing a novel epigenetic regulatory pathway coupling ketone metabolism with histone methylations and that PGC1α is an important target of its epigenetic effects.

In addition to the direct regulation of histone post-translational modifications by ketone bodies, many metabolites of the ketone-TCA cycle pathway can also regulate histone PTMs (Abdul Kadir et al., 2020). These mechanisms include the regulation of citric acid signaling to histone acetyltransferases (HATs) (Deb et al., 2017), α-ketoglutaric acid conversion to histone methyltransferases (HMTs) (Karlstaedt et al., 2016), and auxiliary glucose signaling pathways (Medford et al., 2013; Kronlage et al., 2019), such as the hexosamine (UDP-GlcNAc) biosynthesis pathway, which affects histone PTMs through direct O-GlcNAcylation of histones or indirect O-GlcNAcylation of HDAC4. However, the regulatory mechanisms of these histone post-translational modifications in failing hearts require further investigation.

#### 4.4.2 DNA modification

Changes in dietary patterns can modulate the methylation of key metabolic genes, thereby regulating their expression. 5-Methylcytosine (5mC) and 5-hydroxymethylcytosine (5 hmC) are common DNA modifications. Typically, 5mC at gene promoters inversely correlates with gene expression, whereas the relatively understudied 5 hmC, found within gene bodies, usually positively correlates with gene expression.

Early studies linked DNA 5mC alterations to the expression pattern of angiogenesis-related genes involved in the progression of human heart failure (Movassagh et al., 2010). Some changes in DNA methylation in human heart failure are strongly correlated with coordinated alterations in enzymes responsible for the conversion of fatty acid to glucose, which are potentially regulated by DNA methyltransferase 3A (DNMT3A) (Pepin et al., 2019a; Pepin et al., 2019b). Recent evidence suggests that a ketogenic diet with high-fat and low-carbohydrate content can improve DNA methylation-mediated gene expression changes (Kobow et al., 2013). Several studies have shown that β-HB can increase adenosine levels (Lusardi et al., 2015), leading to the formation of S-adenosylhomocysteine (SAH) (Williams-Karnesky et al., 2013), which inhibits DNA methyltransferases and reduces DNA methylation (Williams-Karnesky et al., 2013; Chen et al., 2019a). However, the relationship between ketone metabolism and the pattern of DNA methylation and cardiac gene expression in heart failure is complex (Lother et al., 2021). Therefore, additional research is needed to understand the contribution and adjustment of these pathways.

#### 4.4.3 Non-coding RNA

Like histone PTMs, non-coding RNAs (ncRNAs), including various pathways such as microRNAs (miRNAs), long non-coding RNAs (lncRNAs), and circular RNAs (circRNAs), play a dominant role in epigenetic regulation and have attracted increasing interest in heart failure due to their ability to regulate gene expression in heart failure (Gomes et al., 2020).

In a caloric restriction mouse model, the levels of serum miR-16-5p, miR-196b-5p, and miR-218-5p were increased, and miR-16-5p expression in the heart was also significantly elevated to suppress inflammatory cytokines (Yamada et al., 2020), suggesting that changes in microRNA levels during caloric restriction can maintain immune homeostasis and regulate inflammation. A study in obese volunteers revealed that a ketogenic diet led to changes in the levels of microRNAs associated with antioxidant and anti-inflammatory signaling pathways, but these levels returned to normal after termination of the KD (Cannataro et al., 2019). Despite the multiple regulatory roles of ketones on miRNAs in various cardiovascular diseases, the detailed regulatory effects of ketones in preventing and treating heart failure require further study.

Compared to the expression profiles of mRNAs or miRNAs, lncRNAs are considered sensitive regulatory factors associated with HF and involve numerous regulatory pathways. The strong correlation between lncRNAs and HF makes lncRNAs attractive biomarkers for HF. For example, the HypERlnc is expressed in pericytes and downregulated in the hearts of patients with heart failure (Bischoff et al., 2017). Although circRNAs are expressed and regulate cell proliferation and transformation in vascular smooth muscle cells and endothelial cells (Chen et al., 2017; Zhong et al., 2017), their role in the development of heart failure is still poorly understood. Taken together, lncRNAs and circRNAs are attractive targets for heart failure, but our insights into the molecular pathology mechanisms dominated by lncRNAs and circRNAs have not yet emerged in the prevention and treatment of heart failure based on ketone bodies.

### 4.5 Gut microbiota

The gut microbiota is closely related to the occurrence of cardiovascular diseases, especially the development of heart failure (Mamic et al., 2023; Chen X. et al., 2019). Crawford and colleagues (Masenga et al., 2023) reported that, compared to conventional wild-type mice, germ-free mice had smaller hearts and abnormal myocardial metabolism. However, these differences could be eliminated by transplanting the gut microbiota or by maintaining a 2-week ketogenic diet. This may be due to the unique synthetic enzyme system of microbes, which affects the production and utilization of energy substances such as ketone bodies and subsequently promotes biological effects such as anti-oxidation and anti-inflammation in heart failure. Most studies have focused on the gut microbiota regulating the host’s cardiac physiology and pathology by their metabolites, including regulating heart size and physiological functions through ketone bodies generation (Masenga et al., 2023; Masenga et al., 2022; Chen X. et al., 2023). Additionally, acetate, microbial short chain fatty acid metabolites, acutely lowers heart rate and blood pressure via modulating sympathetic tone, cardiac contractility and the transmission of endothelial GPR41-involved signaling (Crawford et al., 2009; Poll et al., 2021; Natarajan et al., 2016; Hu et al., 2022).

Notably, the composition, diversity, and function of the gut microbiota are also influenced by ketogenic diets (Chen H. C. et al., 2023; Zambrano et al., 2023; Griffin et al., 2017; Tagliabue et al., 2017; Swidsinski et al., 2017), and ketone-induced gut microbiome remodeling is inducible and reproducible (Olson et al., 2018).“Ketogenic microbiota” is defined as the characteristic of the gut microbiota shaped by ketogenic diets (Cabrera-Mulero et al., 2019), and it may be a crucial factor in the effectiveness of ketone therapy for treating diseases. Numerous studies have shown that ketogenic diets can reshape the gut microbiota in humans and rodents; this ketogenic microbiota is essential for the efficacy of therapeutic ketones (Olson et al., 2018; Spinelli and Blackford, 2018; Hampton, 2018). Subjects can be classified as responders or non-responders based on changes in their gut microbiota (Zhang et al., 2018; Sanchez-Quintero et al., 2022), indicating that the effectiveness of ketosis therapy is partly driven by the gut microbiota. The resistance of germ-free animals to the therapeutic effects of ketosis further elucidates the direct correlation between the ketogenic microbiota and the therapeutic efficacy of ketone bodies (Hampton, 2018). In summary, these studies demonstrate that the gut microbiota plays an important mediating role between ketones and host physiology.

### 4.6 Other mechanisms

In addition to the above mechanisms, ketones have also been reported to act as precursors for lipid synthesis rather than as energy substrates, playing a positive role in cardiovascular diseases (Lopaschuk et al., 2021; Grabacka et al., 2016; Hildebrandt et al., 1995). Furthermore, studies have shown that ketones have an endothelium-dependent vasodilatory effect. Clinically, the administration of ketones to patients has been shown to increase vasodilation and blood flow, thereby alleviating symptoms of heart failure (Nielsen et al., 2019). Treatment with (R)-1,3-butanediol can increase the activity of nitric oxide synthase in the resistant arteries of Dahl salt-sensitive rats, although with undesirable side effects (Yurista et al., 2021b). Moreover, mice fed ketogenic diets have also shown increased protein levels of nitric oxide synthase (Ma et al., 2018). These results reveal the multilevel and complex regulatory effects of ketone bodies on the energy metabolism network of the body and suggest that more rigorous research is needed to evaluate the effects of ketone bodies on failing hearts.

Conclusively, the benefits of ketones for a failing heart are not limited to energy metabolism, and exploring the protective mechanisms of ketone bodies is important for further treating heart failure and other diseases.

## 5 Clinical strategies for therapeutic ketosis

Physiologically, the levels of circulating ketones in humans typically range from 0.05 to 0.1 mmol/L. When ketone concentrations exceed 0.5 mmol/L, a state of ketosis is considered to be reached. Fasting or long-term exercise can elevate ketone levels to over 1 mmol/L (Yurista et al., 2021b). For improving heart failure, the dosage range of ketones is crucial, with the optimal therapeutic concentration ranging from 1 to 3 mmol/L, as a concentration higher than 3 mmol/L (considered to indicate acidosis due to ketosis) may have adverse effects on the body (Chu et al., 2021).

**FIGURE 5:**
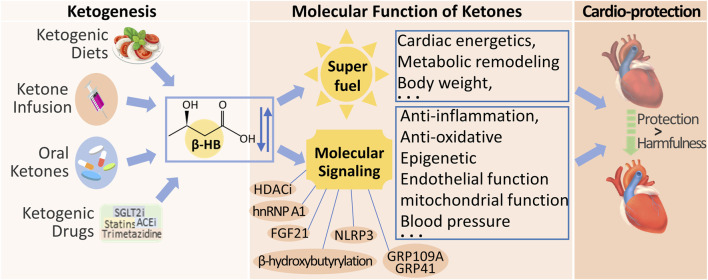
Clinical Strategies for Therapeutic Ketosis and Multiple Signal Effects on Cardiac Function. Ketogenic diets, ketone infusion, oral ketones, and endogenous Ketogenic Drugs are currently available strategies to increase **β-HB** levels in the heart. Beyond its metabolic role, β-HB is also a signaling metabolite to regulate cellular signaling by targeting different biomolecules, for improving cardiac function.

Several methods are currently available to increase ketone body levels in the heart, including ketogenic diets, ketone infusions, and oral ketone supplements. These methods mildly elevate ketone levels in the body, thereby improving heart failure symptoms (Figure 5). Furthermore, as the duration of administration increases, the protective effects of ketones on the cardiovascular system also gradually increase. Table 1 summarizes the effects of different ketogenic methods on the cardiovascular system.

**TABLE 1:** Different ketogenic methods and their cardioprotive effects.

ketogenic methods	objects	Interventions	Major outcomes
Human			
Ketone Infusion	Patients with chronic HFrEF (Nielsen et al., 2019)	β-HB infusion	Ketone body increases energy supply and cardiac output, and decreases systemic vascular resistance in a dose-dependent manner
Oral Ketones	Health populations (Selvaraj et al., 2022)	Ketone ester	
Animals			
Ketogenic Diets	mice with myocardial hypertrophy (Nakamura et al., 2021)	High-fat and low-carb diets	Reduce cardiac hypertrophy and myocardial fibrosis; Improve cardiac systolic dysfunction; Slow down the rational remodeling of heart disease; Reduce left ventricular dilatation; Inhibite cardiomyocyte hypertrophy; Enhance the contractility of cardiomyocytes; Increase the number of mitochondria
Ketogenic Diets	rats with ischemic injury (Al-Zaid et al., 2007)	High-fat and low-carb diets	
Ketone Infusion	dogs with dilated myocardium (Horton et al., 2019)	β-HB infusion	
Oral Ketones	Rats and mice with HF (Yurista et al., 2021a)	Ketone ester drinks	

### 5.1 Ketogenic diets

A ketogenic diet is a dietary pattern consisting of high-fat, low-carbohydrate, and moderate-protein proportions (Gibson et al., 2015). Restricting carbohydrate intake induces a hypoglycemic state, resulting in decreased insulin levels and increased glucagon levels and stimulating lipolysis and ketone production (Puchalska and Crawford, 2017). Animal studies have shown improvements in myocardial hypertrophy and systolic dysfunction in mice with heart failure on a ketogenic diet (Nakamura et al., 2021; McCommis et al., 2020). In a small clinical trial, two patients with left ventricular hypertrophy and heart failure showed significant reductions in heart failure biomarkers and improved cardiac function after 1 year of ketogenic diet therapy (Brambilla et al., 2014). A KD stimulates endogenous ketone production by adjusting the dietary structure and has protective effects on damaged hearts.

The classical ketogenic diet focuses on increased intake of long-chain fats, which may lead to obesity. Therefore, ketogenic diets mainly composed of medium-chain fatty acids are now more commonly used (Mumme and Stonehouse, 2015; Kosinski and Jornayvaz, 2017). Compared with long-chain triglycerides, medium-chain fatty acids can produce ketones more rapidly and have the added benefit of weight reduction. A ketogenic diet is currently emerging as a trend for weight loss, but it is not suitable for long-term use due to poor patient compliance. Prolonged low-carbohydrate diets may increase mortality rates (Seidelmann et al., 2018), and excessive intake of fatty acids can also lead to dyslipidemia, posing significant risks for patients with atherosclerosis and potentially causing non-alcoholic fatty liver and insulin resistance (Kossoff and Hartman, 2012; Zhang W. et al., 2021; Nordmann et al., 2006). Thus, maintaining high ketone levels through a ketogenic diet is not a sustainable long-term solution.

### 5.2 Ketone infusion

Ketone infusion is a method of directly supplementing ketones into the body. Unlike a ketogenic diet, a ketogenic diet does not rely on endogenous fatty acids or glucose and can rapidly increase ketone levels without the process of fatty acid β-oxidation. Studies have shown that ketone infusion can significantly reduce the myocardial infarct size and apoptosis rate in rats with ischemia‒reperfusion injury (Zou et al., 2002). Horton et al. (2019) reported that long-term infusion of β-HB significantly improved cardiac conditions in dogs, increasing the left ventricular ejection fraction (LVEF) and reducing the left ventricular end-diastolic diameter (LVEDD). Moreover, human studies have shown that ketone infusion in heart failure patients can improve cardiac function without increasing myocardial energy consumption when circulating ketone levels reach 3.3 mmol/L (De Jong and Lopaschuk, 2017; Selvaraj et al., 2020; Bedi et al., 2016; Nielsen et al., 2019; Horton et al., 2019).

Compared to ketogenic diets, ketone infusions have fewer drawbacks, are more controllable, and can rapidly elevate ketone levels. However, ketone infusion is not suitable for patients with chronic heart failure due to limitations in the treatment modality, but it is a promising therapeutic option for patients with acute heart failure.

### 5.3 Oral ketones

Oral ketone supplements can be divided into ketone salts and ketone esters, but ketone salts are not suitable for long-term oral consumption due to their high sodium content. In contrast, ketone esters have greater therapeutic significance for chronic heart failure. Ketone esters include (R)-1,3-butanediol and (R)-3-hydroxybutyrate ethyl ester. A study on heart failure mice revealed that ketones can restore ATP levels in damaged hearts and ameliorate heart failure symptoms (Yurista et al., 2021a), indicating that ketones alleviate impaired cardiac function by providing energy to the failing heart. The role of ketone bodies in the treatment of heart failure was further demonstrated by Takahara et al. (2021). Long-term supplementation with ketone esters significantly increases circulating ketone levels, improves myocardial hypertrophy, and significantly increases cardiac output in heart failure mice. Currently, ketone ester trials are mainly conducted in healthy individuals (Selvaraj et al., 2022), with limited research on heart failure patients. Monzo et al. (2021) reported that heart failure patients exhibit increased cardiac uptake and utilization of ketones after taking ketone esters. However, long-term studies on the effects of ketone esters on heart failure are still lacking and require further exploration.

As the most advanced method for supplementing internal ketones, the ketone ester diet is superior to other methods in terms of safety and efficacy, with fewer disadvantages. Although (R)-1,3-butanediol may cause euphoria, dizziness, and gastrointestinal reactions (Shaw et al., 2019), these adverse effects are not common (Stubbs et al., 2019). Therefore, future research can investigate the protective effects of a ketone ester diet on heart failure.

### 5.4 Endogenous Ketogenic Drugs

#### 5.4.1 SGLT2 inhibitors

SGLT2 inhibitors are a novel class of anti-diabetic drugs that work by inhibiting the reabsorption of glucose in the proximal tubules of the kidneys, increasing glucose excretion in the urine, and thereby exerting hypoglycemic effects. SGLT2 inhibitors provide certain cardiovascular protection, among which empagliflozin and dapagliflozin have been approved for the treatment of heart failure (Zannad et al., 2020). The exact mechanisms underlying the cardioprotective effects of SGLT2 inhibitors in heart failure are not fully understood. However, these benefits are unlikely to be solely explained by improved blood glucose control, as other antidiabetic medications have not shown similar protective effects on the damaged heart, and SGLT2 inhibitors have also demonstrated cardioprotective effects in non-diabetic heart failure animals or patients (Yurista et al., 2019; Escobar et al., 2023). Furthermore, SGLT2 receptors are hardly expressed in the heart, suggesting a lesser possibility of direct cardioprotection through receptor‒ligand interactions (Lytvyn et al., 2017).

Currently, many researchers believe that SGLT2 inhibitors exert their cardioprotective effects by increasing ketone bodies. Studies have shown that patients using SGLT2 inhibitors have elevated circulating ketone levels (Ferrannini et al., 2016). The mechanisms by which SGLT2 inhibitors increase ketones may involve two processes: on the one hand, by increasing glucose excretion in the urine and mimicking a state of starvation, SGLT2 inhibitors increase endogenous glucagon levels and decrease the insulin/glucagon ratio by increasing the excretion of glucose in the urine to simulate the starvation state (Wallenius et al., 2022); on the other hand, SGLT2 inhibitors can directly act on pancreatic β-cells to enhance glucagon secretion (Bonner et al., 2015). Previous research has demonstrated that SGLT2 inhibitors can exert cardioprotective effects by increasing ketone metabolism. In a study on non-diabetic myocardial infarction rats, empagliflozin increased circulating ketone levels and upregulated the expression of key enzymes involved in ketone metabolism (Yurista et al., 2019). Another study of myocardial infarction pigs treated with empagliflozin revealed increased cardiac ketone uptake rates and levels of ketone metabolism enzymes, along with decreased glucose uptake rates, indicating that empagliflozin shifts the cardiac energy substrate from glucose to ketones, improving energy deficiency and alleviating heart failure symptoms (Santos-Gallego et al., 2019).

The ideal therapeutic concentration of ketones is between 1 and 3 mmol/L, and SGLT2 inhibitors elevate ketone levels within this range (Chu et al., 2021). Therefore, the mechanism by which SGLT2 inhibitors treat heart failure through increasing ketone levels is highly important.

#### 5.4.2 Other medications

In addition to SGLT2 inhibitors, some drugs have also been observed to alter ketone levels in patients to varying degrees. Statins competitively block the cholesterol synthesis pathway by inhibiting HMG-CoA reductase, thereby increasing the amount of raw materials available for ketone body synthesis and increasing the level of ketone bodies in the body (Tsouli et al., 2008; Clearfield, 2002; von Haehling et al., 2003). However, the effects of statins on heart failure remain highly debated, and to date, there have been no definitive reports of statins improving heart failure outcomes. Two large clinical trials have shown that while rosuvastatin reduces heart failure hospitalization rates, it does not reduce cardiovascular disease mortality (Florkowski et al., 2008; Tavazzi et al., 2008). Some researchers have proposed that statins may cause mitochondrial toxicity, which could worsen heart failure (Okuyama et al., 2015a; Okuyama et al., 2015b). Clinical studies have shown that after 3 months of statin therapy, patients’ ketone levels increase from 0.16 mmol/L to 0.26 mmol/L (Baul et al., 2020), which is far below the ideal therapeutic range for ketones. Although it can be inferred from the mechanism of action that statins may elevate ketone levels, their ability to do so is relatively weak in practical applications, and they do not reach the effective concentration range for ketone-mediated cardiac protection.

Sacubitril/valsartan is a novel drug for heart failure treatment (Abdin et al., 2022; Ghionzoli et al., 2022). Its mechanism of action involves inhibiting neprilysin and angiotensin receptors, increasing the levels of atrial natriuretic peptide and angiotensin, enhancing renin activity, and exerting diuretic, blood pressure-lowering, and peripheral vasodilatory effects, thereby improving pathological remodeling of the damaged heart and relieving heart failure (Docherty et al., 2020; Aimo et al., 2022). Traditional heart failure medications also act on the renin-angiotensin-aldosterone system (RAAS), but their therapeutic effects are far from those of sacubitril/valsartan. Sacubitril/valsartan can increase endogenous glucagon levels, even up to twice the baseline, and glucagon can increase ketone levels (Kjeldsen et al., 2021; Gori et al., 2021). Therefore, sacubitril/valsartan may improve heart failure by increasing ketone levels through elevated glucagon. However, there is currently no research on this hypothesis, and further exploration is needed.

Trimetazidine is an anti-anginal medication that improves cardiac energy metabolism by modulating fatty acid oxidation and exerting cardioprotective effects (Shu et al., 2020; Mahajan and Mahajan, 2020). An experiment confirmed that trimetazidine increased the levels of ketone metabolism-related proteins in heart failure rats and reduced ketone levels after treatment, improving pathological cardiac remodeling (Li H. et al., 2020). Therefore, it is believed that trimetazidine improves heart failure by enhancing the utilization of ketones in heart failure rats. Research on the relationship between trimetazidine and ketones is still relatively limited, but further exploration of the relationship between trimetazidine and ketones, an energy-modulating drug, is important.

#### 5.4.3 Studies in perspectives

Increasing evidence suggests that elevated ketones have beneficial effects on heart failure, improving heart condition and slowing disease progression. However, it is important to note that ketone levels during treatment need to be maintained within a certain range, as both excessively high or low levels may be detrimental. The ketone concentration range maintained by SGLT2 inhibitors can serve as a reference. Currently, research on the role of ketones in improving heart failure is still in its early stages (Supplementary Table S1). Future studies need to explore the protective mechanisms of ketones on the heart, identify safer methods of ketone administration, and assess the long-term safety and efficacy of ketone therapy.

In the realm of drug development, focusing on key enzymes and transporters involved in ketone metabolism, such as BDH1 and SCOT, novel therapeutics could provide more targeted and effective treatment options for heart failure patients by targeting ketone metabolism pathways.

## 6 Conclusion

In conclusion, the cardiovascular protective effects of ketone bodies will be more thoroughly investigated, and ketone bodies have the potential to become an important new field for the treatment of heart failure.

## References

[B1] Abdelmegeed M. A. Kim S. K. Woodcroft K. J. Novak R. F. (2004). Acetoacetate activation of extracellular signal-regulated kinase 1/2 and p38 mitogen-activated protein kinase in primary cultured rat hepatocytes: role of oxidative stress. J. Pharmacol. Exp. Ther. 310 (2), 728–736. 10.1124/jpet.104.066522 15051799

[B2] Abdin A. Schulz M. Riemer U. Haderi B. Wachter R. Laufs U. (2022). Sacubitril/valsartan in heart failure: efficacy and safety in and outside clinical trials. Esc. Heart Fail 9 (6), 3737–3750. 10.1002/ehf2.14097 35921043 PMC9773772

[B3] Abdul Kadir A. Clarke K. Evans R. D. (2020). Cardiac ketone body metabolism. Biochim. Biophys. Acta Mol. Basis Dis. 1866 (6), 165739. 10.1016/j.bbadis.2020.165739 32084511

[B4] Ahmed K. Tunaru S. Offermanns S. (2009). GPR109A, GPR109B and GPR81, a family of hydroxy-carboxylic acid receptors. Trends Pharmacol. Sci. 30 (11), 557–562. 10.1016/j.tips.2009.09.001 19837462

[B5] Aimo A. Castiglione V. Vergaro G. Panichella G. Senni M. Lombardi C. M. (2022). The place of vericiguat in the landscape of treatment for heart failure with reduced ejection fraction. Heart Fail Rev. 27 (4), 1165–1171. 10.1007/s10741-021-10146-1 34291399 PMC9197896

[B6] Al-Zaid N. S. Dashti H. M. Mathew T. C. Juggi J. S. (2007). Low carbohydrate ketogenic diet enhances cardiac tolerance to global ischaemia. Acta Cardiol. 62 (4), 381–389. 10.2143/AC.62.4.2022282 17824299

[B7] Aubert G. Martin O. J. Horton J. L. Lai L. Vega R. B. Leone T. C. (2016). The failing heart relies on ketone bodies as a fuel. Circulation 133 (8), 698–705. 10.1161/CIRCULATIONAHA.115.017355 26819376 PMC4766035

[B8] Aubert G. Vega R. B. Kelly D. P. (2013). Perturbations in the gene regulatory pathways controlling mitochondrial energy production in the failing heart. Biochim. Biophys. Acta 1833 (4), 840–847. 10.1016/j.bbamcr.2012.08.015 22964268 PMC3570640

[B9] Bae H. R. Kim D. H. Park M. H. Lee B. Kim M. J. Lee E. K. (2016). β-Hydroxybutyrate suppresses inflammasome formation by ameliorating endoplasmic reticulum stress via AMPK activation. Oncotarget 7 (41), 66444–66454. 10.18632/oncotarget.12119 27661104 PMC5341812

[B10] Baul P. B. Deepak A. D. Kakkar M. Modi S. (2020). Effect of Atorvastatin on blood ketone levels and glycemic control in patients with type 2 diabetes mellitus: a single arm pilot study. Diabetes Metab. Syndr. 14 (5), 1333–1337. 10.1016/j.dsx.2020.07.020 32755832

[B11] Bedi K. C. Jr. Snyder N. W. Brandimarto J. Aziz M. Mesaros C. Worth A. J. (2016). Evidence for intramyocardial disruption of lipid metabolism and increased myocardial ketone utilization in advanced human heart failure. Circulation 133 (8), 706–716. 10.1161/CIRCULATIONAHA.115.017545 26819374 PMC4779339

[B12] Bischoff F. C. Werner A. John D. Boeckel J. N. Melissari M. T. Grote P. (2017). Identification and functional characterization of hypoxia-induced endoplasmic reticulum stress regulating lncRNA (HypERlnc) in pericytes. Circ. Res. 121 (4), 368–375. 10.1161/CIRCRESAHA.116.310531 28611075

[B13] Bonner C. Kerr-Conte J. Gmyr V. Queniat G. Moerman E. Thevenet J. (2015). Inhibition of the glucose transporter SGLT2 with dapagliflozin in pancreatic alpha cells triggers glucagon secretion. Nat. Med. 21 (5), 512–517. 10.1038/nm.3828 25894829

[B14] Brambilla A. Mannarino S. Pretese R. Gasperini S. Galimberti C. Parini R. (2014). Improvement of cardiomyopathy after high-fat diet in two siblings with glycogen storage disease type III. JIMD Rep. 17, 91–95. 10.1007/8904_2014_343 25308556 PMC4241197

[B15] Braunwald E. (2013). Heart failure. JACC Heart Fail 1 (1), 1–20. 10.1016/j.jchf.2012.10.002 24621794

[B16] Byrne N. J. Soni S. Takahara S. Ferdaoussi M. Al Batran R. Darwesh A. M. (2020). Chronically elevating circulating ketones can reduce cardiac inflammation and blunt the development of heart failure. Circ. Heart Fail 13 (6), e006573. 10.1161/CIRCHEARTFAILURE.119.006573 32493060

[B17] Cabrera-Mulero A. Tinahones A. Bandera B. Moreno-Indias I. Macias-Gonzalez M. Tinahones F. J. (2019). Keto microbiota: a powerful contributor to host disease recovery. Rev. Endocr. Metab. Disord. 20 (4), 415–425. 10.1007/s11154-019-09518-8 31720986 PMC6938789

[B18] Cannataro R. Caroleo M. C. Fazio A. La Torre C. Plastina P. Gallelli L. (2019). Ketogenic diet and microRNAs linked to antioxidant biochemical homeostasis. Antioxidants (Basel) 8 (8), 269. 10.3390/antiox8080269 31382449 PMC6719224

[B19] Chen F. He X. Luan G. Li T. (2019a). Role of DNA methylation and adenosine in ketogenic diet for pharmacoresistant epilepsy: focus on epileptogenesis and associated comorbidities. Front. Neurol. 10, 119. 10.3389/fneur.2019.00119 30863356 PMC6399128

[B20] Chen H. C. Liu Y. W. Chang K. C. Wu Y. W. Chen Y. M. Chao Y. K. (2023b). Gut butyrate-producers confer post-infarction cardiac protection. Nat. Commun. 14 (1), 7249. 10.1038/s41467-023-43167-5 37945565 PMC10636175

[B21] Chen J. Cui L. Yuan J. Zhang Y. Sang H. (2017). Circular RNA WDR77 target FGF-2 to regulate vascular smooth muscle cells proliferation and migration by sponging miR-124. Biochem. Biophys. Res. Commun. 494 (1-2), 126–132. 10.1016/j.bbrc.2017.10.068 29042195

[B22] Chen X. Li H. Y. Hu X. M. Zhang Y. Zhang S. Y. (2019b). Current understanding of gut microbiota alterations and related therapeutic intervention strategies in heart failure. Chin. Med. J. Engl. 132 (15), 1843–1855. 10.1097/CM9.0000000000000330 31306229 PMC6759126

[B23] Chen X. Zhang H. Ren S. Ding Y. Remex N. S. Bhuiyan M. S. (2023a). Gut microbiota and microbiota-derived metabolites in cardiovascular diseases. Chin. Med. J. Engl. 136 (19), 2269–2284. 10.1097/CM9.0000000000002206 37442759 PMC10538883

[B24] Chu Y. Zhang C. Xie M. (2021). Beta-hydroxybutyrate, friend or foe for stressed hearts. Front. Aging 2, 681513. 10.3389/fragi.2021.681513 35309549 PMC8932950

[B25] Cinato M. Andersson L. Miljanovic A. Laudette M. Kunduzova O. Boren J. (2024). Role of perilipins in oxidative stress-implications for cardiovascular disease. Antioxidants (Basel) 13 (2), 209. 10.3390/antiox13020209 38397807 PMC10886189

[B26] Clearfield M. B. (2002). Statins: balancing benefits, efficacy and safety. Expert Opin. Pharmacother. 3 (5), 469–477. 10.1517/14656566.3.5.469 11996626

[B27] Cotter D. G. Schugar R. C. Crawford P. A. (2013). Ketone body metabolism and cardiovascular disease. Am. J. Physiol. Heart Circ. Physiol. 304 (8), H1060–H1076. 10.1152/ajpheart.00646.2012 23396451 PMC3625904

[B28] Crawford P. A. Crowley J. R. Sambandam N. Muegge B. D. Costello E. K. Hamady M. (2009). Regulation of myocardial ketone body metabolism by the gut microbiota during nutrient deprivation. Proc. Natl. Acad. Sci. U. S. A. 106 (27), 11276–11281. 10.1073/pnas.0902366106 19549860 PMC2700149

[B29] Deb D. K. Chen Y. Sun J. Wang Y. Li Y. C. (2017). ATP-citrate lyase is essential for high glucose-induced histone hyperacetylation and fibrogenic gene upregulation in mesangial cells. Am. J. Physiol. Ren. Physiol. 313 (2), F423-F429–F429. 10.1152/ajprenal.00029.2017 28490526

[B30] De Jong K. A. Lopaschuk G. D. (2017). Complex energy metabolic changes in heart failure with preserved ejection fraction and heart failure with reduced ejection fraction. Can. J. Cardiol. 33 (7), 860–871. 10.1016/j.cjca.2017.03.009 28579160

[B31] Deng Y. Xie M. Li Q. Xu X. Ou W. Zhang Y. (2021). Targeting mitochondria-inflammation circuit by β-hydroxybutyrate mitigates HFpEF. Circ. Res. 128 (2), 232–245. 10.1161/CIRCRESAHA.120.317933 33176578

[B32] Docherty K. F. Vaduganathan M. Solomon S. D. McMurray J. J. V. (2020). Sacubitril/valsartan: neprilysin inhibition 5 Years after PARADIGM-HF. JACC Heart Fail 8 (10), 800–810. 10.1016/j.jchf.2020.06.020 33004114 PMC8837825

[B33] Doenst T. Nguyen T. D. Abel E. D. (2013). Cardiac metabolism in heart failure: implications beyond ATP production. Circ. Res. 113 (6), 709–724. 10.1161/CIRCRESAHA.113.300376 23989714 PMC3896379

[B34] Du Z. Shen A. Huang Y. Su L. Lai W. Wang P. (2014). 1H-NMR-based metabolic analysis of human serum reveals novel markers of myocardial energy expenditure in heart failure patients. PLoS One 9 (2), e88102. 10.1371/journal.pone.0088102 24505394 PMC3914925

[B35] Eom G. H. Kook H. (2014). Posttranslational modifications of histone deacetylases: implications for cardiovascular diseases. Pharmacol. Ther. 143 (2), 168–180. 10.1016/j.pharmthera.2014.02.012 24594235

[B36] Escobar C. Pascual-Figal D. Manzano L. Nunez J. Camafort M. (2023). Current role of SLGT2 inhibitors in the management of the whole spectrum of heart failure: focus on dapagliflozin. J. Clin. Med. 12 (21), 6798. 10.3390/jcm12216798 37959263 PMC10649290

[B37] Ferrannini E. Baldi S. Frascerra S. Astiarraga B. Heise T. Bizzotto R. (2016). Shift to fatty substrate utilization in response to sodium-glucose cotransporter 2 inhibition in subjects without diabetes and patients with type 2 diabetes. Diabetes 65 (5), 1190–1195. 10.2337/db15-1356 26861783

[B38] Florkowski C. M. Molyneux S. L. George P. M. (2008). Rosuvastatin in older patients with systolic heart failure. N. Engl. J. Med. 358 (12), 1301; author reply 1301. author reply 1301. 10.1056/NEJMc073536 18354111

[B39] Funada J. Betts T. R. Hodson L. Humphreys S. M. Timperley J. Frayn K. N. (2009). Substrate utilization by the failing human heart by direct quantification using arterio-venous blood sampling. PLoS One 4 (10), e7533. 10.1371/journal.pone.0007533 19844574 PMC2760135

[B40] Gambardella J. Jankauskas S. S. Kansakar U. Varzideh F. Avvisato R. Prevete N. (2023). Ketone bodies rescue mitochondrial dysfunction via epigenetic remodeling. JACC Basic Transl. Sci. 8 (9), 1123–1137. 10.1016/j.jacbts.2023.03.014 37791311 PMC10543927

[B41] Ghionzoli N. Gentile F. Del Franco A. M. Castiglione V. Aimo A. Giannoni A. (2022). Current and emerging drug targets in heart failure treatment. Heart Fail Rev. 27 (4), 1119–1136. 10.1007/s10741-021-10137-2 34273070 PMC9197912

[B42] Gibson A. A. Seimon R. V. Lee C. M. Ayre J. Franklin J. Markovic T. P. (2015). Do ketogenic diets really suppress appetite? A systematic review and meta-analysis. Obes. Rev. 16 (1), 64–76. 10.1111/obr.12230 25402637

[B43] Gomes C. P. C. Schroen B. Kuster G. M. Robinson E. L. Ford K. Squire I. B. (2020). Regulatory RNAs in heart failure. Circulation 141 (4), 313–328. 10.1161/CIRCULATIONAHA.119.042474 31986093 PMC7012349

[B44] Gori M. Januzzi J. L. D'Elia E. Lorini F. L. Senni M. (2021). Rationale for and practical use of sacubitril/valsartan in the patient's journey with heart failure and reduced ejection fraction. Card. Fail Rev. 7, e06. 10.15420/cfr.2020.25 33889425 PMC8054374

[B45] Grabacka M. Pierzchalska M. Dean M. Reiss K. (2016). Regulation of ketone body metabolism and the role of PPARα. Int. J. Mol. Sci. 17 (12), 2093. 10.3390/ijms17122093 27983603 PMC5187893

[B46] Griffin N. W. Ahern P. P. Cheng J. Heath A. C. Ilkayeva O. Newgard C. B. (2017). Prior dietary practices and connections to a human gut microbial metacommunity alter responses to diet interventions. Cell. Host Microbe 21 (1), 84–96. 10.1016/j.chom.2016.12.006 28041931 PMC5234936

[B47] Gupta A. Akki A. Wang Y. Leppo M. K. Chacko V. P. Foster D. B. (2012). Creatine kinase-mediated improvement of function in failing mouse hearts provides causal evidence the failing heart is energy starved. J. Clin. Investig. 122 (1), 291–302. 10.1172/JCI57426 22201686 PMC3248286

[B48] Haces M. L. Hernandez-Fonseca K. Medina-Campos O. N. Montiel T. Pedraza-Chaverri J. Massieu L. (2008). Antioxidant capacity contributes to protection of ketone bodies against oxidative damage induced during hypoglycemic conditions. Exp. Neurol. 211 (1), 85–96. 10.1016/j.expneurol.2007.12.029 18339375

[B49] Hampton T. (2018). Gut microbes may account for the anti-seizure effects of the ketogenic diet. JAMA 320 (13), 1307. 10.1001/jama.2017.12865 30285157

[B50] He Y. Cheng X. Zhou T. Li D. Peng J. Xu Y. (2023). β-Hydroxybutyrate as an epigenetic modifier: underlying mechanisms and implications. Heliyon 9 (11), e21098. 10.1016/j.heliyon.2023.e21098 37928021 PMC10623287

[B51] Hildebrandt L. A. Spennetta T. Elson C. Shrago E. (1995). Utilization and preferred metabolic pathway of ketone bodies for lipid synthesis by isolated rat hepatoma cells. Am. J. Physiol. 269 (1 Pt 1), C22–C27. 10.1152/ajpcell.1995.269.1.C22 7631749

[B52] Ho K. L. Zhang L. Wagg C. Al Batran R. Gopal K. Levasseur J. (2019). Increased ketone body oxidation provides additional energy for the failing heart without improving cardiac efficiency. Cardiovasc Res. 115 (11), 1606–1616. 10.1093/cvr/cvz045 30778524 PMC6704391

[B53] Horton J. L. Davidson M. T. Kurishima C. Vega R. B. Powers J. C. Matsuura T. R. (2019). The failing heart utilizes 3-hydroxybutyrate as a metabolic stress defense. JCI Insight 4 (4). 10.1172/jci.insight.124079 PMC6478419 30668551

[B54] Hu T. Wu Q. Yao Q. Jiang K. Yu J. Tang Q. (2022). Short-chain fatty acid metabolism and multiple effects on cardiovascular diseases. Ageing Res. Rev. 81, 101706. 10.1016/j.arr.2022.101706 35932976

[B55] Huynh K. (2016). Heart failure: ketone bodies as fuel in heart failure. Nat. Rev. Cardiol. 13 (3), 122–123. 10.1038/nrcardio.2016.22 26864914

[B56] Jain S. K. Kannan K. Lim G. McVie R. Bocchini J. A. Jr. (2002). Hyperketonemia increases tumor necrosis factor-alpha secretion in cultured U937 monocytes and Type 1 diabetic patients and is apparently mediated by oxidative stress and cAMP deficiency. Diabetes 51 (7), 2287–2293. 10.2337/diabetes.51.7.2287 12086962

[B57] Janardhan A. Chen J. Crawford P. A. (2011). Altered systemic ketone body metabolism in advanced heart failure. Tex Heart Inst. J. 38 (5), 533–538. 22163128 PMC3231554

[B58] Jeong M. Y. Lin Y. H. Wennersten S. A. Demos-Davies K. M. Cavasin M. A. Mahaffey J. H. (2018). Histone deacetylase activity governs diastolic dysfunction through a nongenomic mechanism. Sci. Transl. Med. 10 (427), eaao0144. 10.1126/scitranslmed.aao0144 29437146 PMC5908215

[B59] Kanikarla-Marie P. Jain S. K. (2015). Hyperketonemia (acetoacetate) upregulates NADPH oxidase 4 and elevates oxidative stress, ICAM-1, and monocyte adhesivity in endothelial cells. Cell. Physiol. Biochem. 35 (1), 364–373. 10.1159/000369702 25591777 PMC4309197

[B60] Karlstaedt A. Zhang X. Vitrac H. Harmancey R. Vasquez H. Wang J. H. (2016). Oncometabolite d-2-hydroxyglutarate impairs α-ketoglutarate dehydrogenase and contractile function in rodent heart. Proc. Natl. Acad. Sci. U. S. A. 113 (37), 10436–10441. 10.1073/pnas.1601650113 27582470 PMC5027422

[B61] Kashiwaya Y. Takeshima T. Mori N. Nakashima K. Clarke K. Veech R. L. (2000). D-beta-hydroxybutyrate protects neurons in models of Alzheimer's and Parkinson's disease. Proc. Natl. Acad. Sci. U. S. A. 97 (10), 5440–5444. 10.1073/pnas.97.10.5440 10805800 PMC25847

[B62] Keceli G. Gupta A. Sourdon J. Gabr R. Schar M. Dey S. (2022). Mitochondrial creatine kinase attenuates pathologic remodeling in heart failure. Circ. Res. 130 (5), 741–759. 10.1161/CIRCRESAHA.121.319648 35109669 PMC8897235

[B63] Keon C. A. Tuschiya N. Kashiwaya Y. Sato K. Clarke K. Radda G. K. (1995). Substrate dependence of the mitochondrial energy status in the isolated working rat heart. Biochem. Soc. Trans. 23 (2), 307S. 10.1042/bst023307s 7672336

[B64] Khan D. Sarikhani M. Dasgupta S. Maniyadath B. Pandit A. S. Mishra S. (2018). SIRT6 deacetylase transcriptionally regulates glucose metabolism in heart. J. Cell. Physiol. 233 (7), 5478–5489. 10.1002/jcp.26434 29319170

[B65] Kim D. H. Park M. H. Ha S. Bang E. J. Lee Y. Lee A. K. (2019). Anti-inflammatory action of β-hydroxybutyrate via modulation of PGC-1α and FoxO1, mimicking calorie restriction. Aging (Albany NY) 11 (4), 1283–1304. 10.18632/aging.101838 30811347 PMC6402511

[B66] Kimura I. Inoue D. Maeda T. Hara T. Ichimura A. Miyauchi S. (2011). Short-chain fatty acids and ketones directly regulate sympathetic nervous system via G protein-coupled receptor 41 (GPR41). Proc. Natl. Acad. Sci. U. S. A. 108 (19), 8030–8035. 10.1073/pnas.1016088108 21518883 PMC3093469

[B67] Kjeldsen S. A. S. Hansen L. H. Esser N. Mongovin S. Winther-Sorensen M. Galsgaard K. D. (2021). Neprilysin inhibition increases glucagon levels in humans and mice with potential effects on amino acid metabolism. J. Endocr. Soc. 5 (9), bvab084. 10.1210/jendso/bvab084 34337276 PMC8317634

[B68] Kobow K. Kaspi A. Harikrishnan K. N. Kiese K. Ziemann M. Khurana I. (2013). Deep sequencing reveals increased DNA methylation in chronic rat epilepsy. Acta Neuropathol. 126 (5), 741–756. 10.1007/s00401-013-1168-8 24005891 PMC3825532

[B69] Kolb H. Kempf K. Rohling M. Lenzen-Schulte M. Schloot N. C. Martin S. (2021). Ketone bodies: from enemy to friend and guardian angel. BMC Med. 19 (1), 313. 10.1186/s12916-021-02185-0 34879839 PMC8656040

[B70] Kolwicz S. C. Jr. Airhart S. Tian R. (2016). Ketones step to the plate: a game changer for metabolic remodeling in heart failure? Circulation 133 (8), 689–691. 10.1161/CIRCULATIONAHA.116.021230 26819375 PMC4826559

[B71] Kosinski C. Jornayvaz F. R. (2017). Effects of ketogenic diets on cardiovascular risk factors: evidence from animal and human studies. Nutrients 9 (5), 517. 10.3390/nu9050517 28534852 PMC5452247

[B72] Kossoff E. H. Hartman A. L. (2012). Ketogenic diets: new advances for metabolism-based therapies. Curr. Opin. Neurol. 25 (2), 173–178. 10.1097/WCO.0b013e3283515e4a 22322415 PMC4002181

[B73] Krebs P. Fan W. Chen Y. H. Tobita K. Downes M. R. Wood M. R. (2011). Lethal mitochondrial cardiomyopathy in a hypomorphic Med30 mouse mutant is ameliorated by ketogenic diet. Proc. Natl. Acad. Sci. U. S. A. 108 (49), 19678–19682. 10.1073/pnas.1117835108 22106289 PMC3241770

[B74] Kronlage M. Dewenter M. Grosso J. Fleming T. Oehl U. Lehmann L. H. (2019). O-GlcNAcylation of histone deacetylase 4 protects the diabetic heart from failure. Circulation 140 (7), 580–594. 10.1161/CIRCULATIONAHA.117.031942 31195810

[B75] Li C. Huang J. Chen X. Yan Y. Li L. Zhao W. (2022). Transcriptome analysis reveals that NEFA and β-hydroxybutyrate induce oxidative stress and inflammatory response in bovine mammary epithelial cells. Metabolites 12 (11), 1060. 10.3390/metabo12111060 36355143 PMC9696823

[B76] Li H. Ma Z. Zhai Y. Lv C. Yuan P. Zhu F. (2020b). Trimetazidine ameliorates myocardial metabolic remodeling in isoproterenol-induced rats through regulating ketone body metabolism via activating AMPK and PPAR α. Front. Pharmacol. 11, 1255. 10.3389/fphar.2020.01255 32922293 PMC7457052

[B77] Li P. Ge J. Li H. (2020a). Lysine acetyltransferases and lysine deacetylases as targets for cardiovascular disease. Nat. Rev. Cardiol. 17 (2), 96–115. 10.1038/s41569-019-0235-9 31350538

[B78] Li Z. Li X. Lin S. Chen Y. Ma S. Fu Y. (2017). Nicotinic acid receptor GPR109A exerts anti-inflammatory effects through inhibiting the akt/mTOR signaling pathway in MIN6 pancreatic β cells. Ann. Clin. Lab. Sci. 47 (6), 729–737. 29263047

[B79] Liu K. Li F. Sun Q. Lin N. Han H. You K. (2019). p53 β-hydroxybutyrylation attenuates p53 activity. Cell. Death Dis. 10 (3), 243. 10.1038/s41419-019-1463-y 30858356 PMC6411878

[B80] Liu Z. Y. Song K. Tu B. Lin L. C. Sun H. Zhou Y. (2023). Crosstalk between oxidative stress and epigenetic marks: new roles and therapeutic implications in cardiac fibrosis. Redox Biol. 65, 102820. 10.1016/j.redox.2023.102820 37482041 PMC10369469

[B81] Lommi J. Kupari M. Koskinen P. Naveri H. Leinonen H. Pulkki K. (1996). Blood ketone bodies in congestive heart failure. J. Am. Coll. Cardiol. 28 (3), 665–672. 10.1016/0735-1097(96)00214-8 8772754

[B82] Lopaschuk G. D. (2017). Metabolic modulators in heart disease: past, present, and future. Can. J. Cardiol. 33 (7), 838–849. 10.1016/j.cjca.2016.12.013 28279520

[B83] Lopaschuk G. D. Karwi Q. G. Tian R. Wende A. R. Abel E. D. (2021). Cardiac energy metabolism in heart failure. Circ. Res. 128 (10), 1487–1513. 10.1161/CIRCRESAHA.121.318241 33983836 PMC8136750

[B84] Lopaschuk G. D. Ussher J. R. (2016). Evolving concepts of myocardial energy metabolism: more than just fats and carbohydrates. Circ. Res. 119 (11), 1173–1176. 10.1161/CIRCRESAHA.116.310078 28051784

[B85] Lopaschuk G. D. Ussher J. R. Folmes C. D. Jaswal J. S. Stanley W. C. (2010). Myocardial fatty acid metabolism in health and disease. Physiol. Rev. 90 (1), 207–258. 10.1152/physrev.00015.2009 20086077

[B86] Lother A. Bondareva O. Saadatmand A. R. Pollmeier L. Hardtner C. Hilgendorf I. (2021). Diabetes changes gene expression but not DNA methylation in cardiac cells. J. Mol. Cell. Cardiol. 151, 74–87. 10.1016/j.yjmcc.2020.11.004 33197445

[B87] Lusardi T. A. Akula K. K. Coffman S. Q. Ruskin D. N. Masino S. A. Boison D. (2015). Ketogenic diet prevents epileptogenesis and disease progression in adult mice and rats. Neuropharmacology 99, 500–509. 10.1016/j.neuropharm.2015.08.007 26256422 PMC4655189

[B88] Lytvyn Y. Bjornstad P. Udell J. A. Lovshin J. A. Cherney D. Z. I. (2017). Sodium glucose cotransporter-2 inhibition in heart failure: potential mechanisms, clinical applications, and summary of clinical trials. Circulation 136 (17), 1643–1658. 10.1161/CIRCULATIONAHA.117.030012 29061576 PMC5846470

[B89] Ma D. Wang A. C. Parikh I. Green S. J. Hoffman J. D. Chlipala G. (2018). Ketogenic diet enhances neurovascular function with altered gut microbiome in young healthy mice. Sci. Rep. 8 (1), 6670. 10.1038/s41598-018-25190-5 29703936 PMC5923270

[B90] Mahajan S. Mahajan A. U. (2020). Current clinical evidence of trimetazidine in the management of heart disease in patients with diabetes. J. Assoc. Physicians India 68 (11), 46–50. 33187037

[B91] Mamic P. Snyder M. Tang W. H. W. (2023). Gut microbiome-based management of patients with heart failure: JACC review topic of the week. J. Am. Coll. Cardiol. 81 (17), 1729–1739. 10.1016/j.jacc.2023.02.045 37100490

[B92] Mascolo A. di Mauro G. Cappetta D. De Angelis A. Torella D. Urbanek K. (2022). Current and future therapeutic perspective in chronic heart failure. Pharmacol. Res. 175, 106035. 10.1016/j.phrs.2021.106035 34915125

[B93] Masenga S. K. Hamooya B. Hangoma J. Hayumbu V. Ertuglu L. A. Ishimwe J. (2022). Recent advances in modulation of cardiovascular diseases by the gut microbiota. J. Hum. Hypertens. 36 (11), 952–959. 10.1038/s41371-022-00698-6 35469059 PMC9649420

[B94] Masenga S. K. Povia J. P. Lwiindi P. C. Kirabo A. (2023). Recent advances in microbiota-associated metabolites in heart failure. Biomedicines 11 (8), 2313. 10.3390/biomedicines11082313 37626809 PMC10452327

[B95] Mayr M. Yusuf S. Weir G. Chung Y. L. Mayr U. Yin X. (2008). Combined metabolomic and proteomic analysis of human atrial fibrillation. J. Am. Coll. Cardiol. 51 (5), 585–594. 10.1016/j.jacc.2007.09.055 18237690

[B96] McCommis K. S. Kovacs A. Weinheimer C. J. Shew T. M. Koves T. R. Ilkayeva O. R. (2020). Nutritional modulation of heart failure in mitochondrial pyruvate carrier-deficient mice. Nat. Metab. 2 (11), 1232–1247. 10.1038/s42255-020-00296-1 33106690 PMC7957960

[B97] McKinsey T. A. (2012). Therapeutic potential for HDAC inhibitors in the heart. Annu. Rev. Pharmacol. Toxicol. 52, 303–319. 10.1146/annurev-pharmtox-010611-134712 21942627

[B98] Medford H. M. Porter K. Marsh S. A. (2013). Immediate effects of a single exercise bout on protein O-GlcNAcylation and chromatin regulation of cardiac hypertrophy. Am. J. Physiol. Heart Circ. Physiol. 305 (1), H114–H123. 10.1152/ajpheart.00135.2013 23624624 PMC3727102

[B99] Menzies K. J. Zhang H. Katsyuba E. Auwerx J. (2016). Protein acetylation in metabolism - metabolites and cofactors. Nat. Rev. Endocrinol. 12 (1), 43–60. 10.1038/nrendo.2015.181 26503676

[B100] Milder J. Patel M. (2012). Modulation of oxidative stress and mitochondrial function by the ketogenic diet. Epilepsy Res. 100 (3), 295–303. 10.1016/j.eplepsyres.2011.09.021 22078747 PMC3322307

[B101] Monzo L. Sedlacek K. Hromanikova K. Tomanova L. Borlaug B. A. Jabor A. (2021). Myocardial ketone body utilization in patients with heart failure: the impact of oral ketone ester. Metabolism 115, 154452. 10.1016/j.metabol.2020.154452 33248064

[B102] Movassagh M. Choy M. K. Goddard M. Bennett M. R. Down T. A. Foo R. S. (2010). Differential DNA methylation correlates with differential expression of angiogenic factors in human heart failure. PLoS One 5 (1), e8564. 10.1371/journal.pone.0008564 20084101 PMC2797324

[B103] Mullens W. Verbrugge F. H. Nijst P. Tang W. H. W. (2017). Renal sodium avidity in heart failure: from pathophysiology to treatment strategies. Eur. Heart J. 38 (24), 1872–1882. 10.1093/eurheartj/ehx035 28329085

[B104] Mumme K. Stonehouse W. (2015). Effects of medium-chain triglycerides on weight loss and body composition: a meta-analysis of randomized controlled trials. J. Acad. Nutr. Diet. 115 (2), 249–263. 10.1016/j.jand.2014.10.022 25636220

[B105] Murashige D. Jang C. Neinast M. Edwards J. J. Cowan A. Hyman M. C. (2020). Comprehensive quantification of fuel use by the failing and nonfailing human heart. Science 370 (6514), 364–368. 10.1126/science.abc8861 33060364 PMC7871704

[B106] Nagao M. Toh R. Irino Y. Mori T. Nakajima H. Hara T. (2016). β-Hydroxybutyrate elevation as a compensatory response against oxidative stress in cardiomyocytes. Biochem. Biophys. Res. Commun. 475 (4), 322–328. 10.1016/j.bbrc.2016.05.097 27216458

[B107] Nakamura M. Odanovic N. Nakada Y. Dohi S. Zhai P. Ivessa A. (2021). Dietary carbohydrates restriction inhibits the development of cardiac hypertrophy and heart failure. Cardiovasc Res. 117 (11), 2365–2376. 10.1093/cvr/cvaa298 33070172 PMC8861266

[B108] Natarajan N. Hori D. Flavahan S. Steppan J. Flavahan N. A. Berkowitz D. E. (2016). Microbial short chain fatty acid metabolites lower blood pressure via endothelial G protein-coupled receptor 41. Physiol. Genomics 48 (11), 826–834. 10.1152/physiolgenomics.00089.2016 27664183 PMC6223570

[B109] Newman J. C. Verdin E. (2014). Ketone bodies as signaling metabolites. Trends Endocrinol. Metab. 25 (1), 42–52. 10.1016/j.tem.2013.09.002 24140022 PMC4176946

[B110] Nielsen R. Moller N. Gormsen L. C. Tolbod L. P. Hansson N. H. Sorensen J. (2019). Cardiovascular effects of treatment with the ketone body 3-hydroxybutyrate in chronic heart failure patients. Circulation 139 (18), 2129–2141. 10.1161/CIRCULATIONAHA.118.036459 30884964 PMC6493702

[B111] Nordmann A. J. Nordmann A. Briel M. Keller U. Yancy W. S. Jr. Brehm B. J. (2006). Effects of low-carbohydrate vs low-fat diets on weight loss and cardiovascular risk factors: a meta-analysis of randomized controlled trials. Arch. Intern Med. 166 (3), 285–293. 10.1001/archinte.166.3.285 16476868

[B112] Offermanns S. Colletti S. L. Lovenberg T. W. Semple G. Wise A. Ap I. J. (2011). International union of basic and clinical pharmacology. LXXXII: nomenclature and classification of hydroxy-carboxylic acid receptors (GPR81, GPR109A, and GPR109B). Pharmacol. Rev. 63 (2), 269–290. 10.1124/pr.110.003301 21454438

[B113] Oka S. I. Tang F. Chin A. Ralda G. Xu X. Hu C. (2021). β-Hydroxybutyrate, a ketone body, potentiates the antioxidant defense via thioredoxin 1 upregulation in cardiomyocytes. Antioxidants (Basel) 10 (7), 1153. 10.3390/antiox10071153 34356388 PMC8301070

[B114] Okuyama H. Langsjoen P. H. Hamazaki T. (2015b). Erratum. Correction to: statins stimulate atherosclerosis and heart failure: pharmacological mechanisms. Expert Rev. Clin. Pharmacol. 8 (4), 503–505. 10.1586/17512433.2015.1055111 26041341

[B115] Okuyama H. Langsjoen P. H. Hamazaki T. Ogushi Y. Hama R. Kobayashi T. (2015a). Statins stimulate atherosclerosis and heart failure: pharmacological mechanisms. Expert Rev. Clin. Pharmacol. 8 (2), 189–199. 10.1586/17512433.2015.1011125 25655639

[B116] Olson C. A. Vuong H. E. Yano J. M. Liang Q. Y. Nusbaum D. J. Hsiao E. Y. (2018). The gut microbiota mediates the anti-seizure effects of the ketogenic diet. Cell. 173 (7), 1728–1741. 10.1016/j.cell.2018.04.027 29804833 PMC6003870

[B117] Pelletier A. Coderre L. (2007). Ketone bodies alter dinitrophenol-induced glucose uptake through AMPK inhibition and oxidative stress generation in adult cardiomyocytes. Am. J. Physiol. Endocrinol. Metab. 292 (5), E1325–E1332. 10.1152/ajpendo.00186.2006 17227964

[B118] Pepin M. E. Drakos S. Ha C. M. Tristani-Firouzi M. Selzman C. H. Fang J. C. (2019a). DNA methylation reprograms cardiac metabolic gene expression in end-stage human heart failure. Am. J. Physiol. Heart Circ. Physiol. 317 (4), H674-H684–H684. 10.1152/ajpheart.00016.2019 31298559 PMC6843013

[B119] Pepin M. E. Ha C. M. Crossman D. K. Litovsky S. H. Varambally S. Barchue J. P. (2019b). Genome-wide DNA methylation encodes cardiac transcriptional reprogramming in human ischemic heart failure. Lab. Investig. 99 (3), 371–386. 10.1038/s41374-018-0104-x 30089854 PMC6515060

[B120] Poll B. G. Xu J. Jun S. Sanchez J. Zaidman N. A. He X. (2021). Acetate, a short-chain fatty acid, acutely lowers heart rate and cardiac contractility along with blood pressure. J. Pharmacol. Exp. Ther. 377 (1), 39–50. 10.1124/jpet.120.000187 33414131 PMC7985618

[B121] Ponikowski P. Voors A. A. Anker S. D. Bueno H. Cleland J. G. Coats A. J. (2016). 2016 ESC Guidelines for the diagnosis and treatment of acute and chronic heart failure: the Task Force for the diagnosis and treatment of acute and chronic heart failure of the European Society of Cardiology (ESC). Developed with the special contribution of the Heart Failure Association (HFA) of the ESC. Eur. J. Heart Fail 18 (8), 891–975. 10.1002/ejhf.592 27207191

[B122] Puchalska P. Crawford P. A. (2017). Multi-dimensional roles of ketone bodies in fuel metabolism, signaling, and therapeutics. Cell. Metab. 25 (2), 262–284. 10.1016/j.cmet.2016.12.022 28178565 PMC5313038

[B123] Qian N. Wang Y. (2020). Ketone body metabolism in diabetic and non-diabetic heart failure. Heart Fail Rev. 25 (5), 817–822. 10.1007/s10741-019-09857-3 31612363

[B124] Rojas-Morales P. Tapia E. Pedraza-Chaverri J. (2016). beta-Hydroxybutyrate: a signaling metabolite in starvation response? Cell. Signal 28 (8), 917–923. 10.1016/j.cellsig.2016.04.005 27083590

[B125] Sanchez-Quintero M. J. Delgado J. Medina-Vera D. Becerra-Munoz V. M. Queipo-Ortuno M. I. Estevez M. (2022). Beneficial effects of essential oils from the mediterranean diet on gut microbiota and their metabolites in ischemic heart disease and type-2 diabetes mellitus. Nutrients 14 (21), 4650. 10.3390/nu14214650 36364913 PMC9657080

[B126] Santos-Gallego C. G. Requena-Ibanez J. A. San Antonio R. Ishikawa K. Watanabe S. Picatoste B. (2019). Empagliflozin ameliorates adverse left ventricular remodeling in nondiabetic heart failure by enhancing myocardial energetics. J. Am. Coll. Cardiol. 73 (15), 1931–1944. 10.1016/j.jacc.2019.01.056 30999996

[B127] Schugar R. C. Moll A. R. Andre d'Avignon D. Weinheimer C. J. Kovacs A. Crawford P. A. (2014). Cardiomyocyte-specific deficiency of ketone body metabolism promotes accelerated pathological remodeling. Mol. Metab. 3 (7), 754–769. 10.1016/j.molmet.2014.07.010 25353003 PMC4209361

[B128] Seidelmann S. B. Claggett B. Cheng S. Henglin M. Shah A. Steffen L. M. (2018). Dietary carbohydrate intake and mortality: a prospective cohort study and meta-analysis. Lancet Public Health 3 (9), e419–e428. 10.1016/S2468-2667(18)30135-X 30122560 PMC6339822

[B129] Selvaraj S. Hu R. Vidula M. K. Dugyala S. Tierney A. Ky B. (2022). Acute echocardiographic effects of exogenous ketone administration in healthy participants. J. Am. Soc. Echocardiogr. 35 (3), 305–311. 10.1016/j.echo.2021.10.017 34798244 PMC8901445

[B130] Selvaraj S. Kelly D. P. Margulies K. B. (2020). Implications of altered ketone metabolism and therapeutic ketosis in heart failure. Circulation 141 (22), 1800–1812. 10.1161/CIRCULATIONAHA.119.045033 32479196 PMC7304522

[B131] Shaw D. M. Merien F. Braakhuis A. Plews D. Laursen P. Dulson D. K. (2019). The effect of 1,3-butanediol on cycling time-trial performance. Int. J. Sport Nutr. Exerc Metab. 29 (5), 466–473. 10.1123/ijsnem.2018-0284 30632425

[B132] Shi X. Qiu H. (2022). New insights into energy substrate utilization and metabolic remodeling in cardiac physiological adaption. Front. Physiol. 13, 831829. 10.3389/fphys.2022.831829 35283773 PMC8914108

[B133] Shimazu T. Hirschey M. D. Newman J. He W. Shirakawa K. Le Moan N. (2013). Suppression of oxidative stress by β-hydroxybutyrate, an endogenous histone deacetylase inhibitor. Science 339 (6116), 211–214. 10.1126/science.1227166 23223453 PMC3735349

[B134] Shu H. Peng Y. Hang W. Zhou N. Wang D. W. (2020). Trimetazidine in heart failure. Front. Pharmacol. 11, 569132. 10.3389/fphar.2020.569132 33597865 PMC7883591

[B135] Snorek M. Hodyc D. Sedivy V. Durisova J. Skoumalova A. Wilhelm J. (2012). Short-term fasting reduces the extent of myocardial infarction and incidence of reperfusion arrhythmias in rats. Physiol. Res. 61 (6), 567–574. 10.33549/physiolres.932338 23098657

[B136] Spinelli E. Blackford R. (2018). Gut microbiota, the ketogenic diet and epilepsy. Pediatr. Neurol. Briefs 32, 10. 10.15844/pedneurbriefs-32-10 30275670 PMC6149163

[B137] Stanley W. C. Meadows S. R. Kivilo K. M. Roth B. A. Lopaschuk G. D. (2003). beta-Hydroxybutyrate inhibits myocardial fatty acid oxidation *in vivo* independent of changes in malonyl-CoA content. Am. J. Physiol. Heart Circ. Physiol. 285 (4), H1626–H1631. 10.1152/ajpheart.00332.2003 12969881

[B138] Stubbs B. J. Cox P. J. Kirk T. Evans R. D. Clarke K. (2019). Gastrointestinal effects of exogenous ketone drinks are infrequent, mild, and vary according to ketone compound and dose. Int. J. Sport Nutr. Exerc Metab. 29 (6), 596–603. 10.1123/ijsnem.2019-0014 31034254

[B139] Sun H. Olson K. C. Gao C. Prosdocimo D. A. Zhou M. Wang Z. (2016). Catabolic defect of branched-chain amino acids promotes heart failure. Circulation 133 (21), 2038–2049. 10.1161/CIRCULATIONAHA.115.020226 27059949 PMC4879058

[B140] Swidsinski A. Dorffel Y. Loening-Baucke V. Gille C. Goktas O. Reisshauer A. (2017). Reduced mass and diversity of the colonic microbiome in patients with multiple sclerosis and their improvement with ketogenic diet. Front. Microbiol. 8, 1141. 10.3389/fmicb.2017.01141 28702003 PMC5488402

[B141] Taegtmeyer H. (2016). Failing heart and starving brain: ketone bodies to the rescue. Circulation 134 (4), 265–266. 10.1161/CIRCULATIONAHA.116.022141 27462050 PMC6818087

[B142] Tagliabue A. Ferraris C. Uggeri F. Trentani C. Bertoli S. de Giorgis V. (2017). Short-term impact of a classical ketogenic diet on gut microbiota in GLUT1 Deficiency Syndrome: a 3-month prospective observational study. Clin. Nutr. ESPEN 17, 33–37. 10.1016/j.clnesp.2016.11.003 28361745

[B143] Takahara S. Soni S. Phaterpekar K. Kim T. T. Maayah Z. H. Levasseur J. L. (2021). Chronic exogenous ketone supplementation blunts the decline of cardiac function in the failing heart. Esc. Heart Fail 8 (6), 5606–5612. 10.1002/ehf2.13634 34617412 PMC8712827

[B144] Tang W. H. W. Kiang A. (2020). Acute cardiorenal syndrome in heart failure: from dogmas to advances. Curr. Cardiol. Rep. 22 (11), 143. 10.1007/s11886-020-01384-0 32910296

[B145] Tavazzi L. Maggioni A. P. Marchioli R. Barlera S. Franzosi M. G. Latini R. (2008). Effect of rosuvastatin in patients with chronic heart failure (the GISSI-HF trial): a randomised, double-blind, placebo-controlled trial. Lancet 372 (9645), 1231–1239. 10.1016/S0140-6736(08)61240-4 18757089

[B146] Tian W. Wei T. Li B. Wang Z. Zhang N. Xie G. (2014). Pathway of programmed cell death and oxidative stress induced by β-hydroxybutyrate in dairy cow abomasum smooth muscle cells and in mouse gastric smooth muscle. PLoS One 9 (5), e96775. 10.1371/journal.pone.0096775 24801711 PMC4011855

[B147] Tsouli S. G. Liberopoulos E. N. Goudevenos J. A. Mikhailidis D. P. Elisaf M. S. (2008). Should a statin be prescribed to every patient with heart failure? Heart Fail Rev. 13 (2), 211–225. 10.1007/s10741-007-9041-2 17694432

[B148] Uchihashi M. Hoshino A. Okawa Y. Ariyoshi M. Kaimoto S. Tateishi S. (2017). Cardiac-specific Bdh1 overexpression ameliorates oxidative stress and cardiac remodeling in pressure overload-induced heart failure. Circ. Heart Fail 10 (12), e004417. 10.1161/CIRCHEARTFAILURE.117.004417 29242353

[B149] Ussher J. R. Elmariah S. Gerszten R. E. Dyck J. R. (2016). The emerging role of metabolomics in the diagnosis and prognosis of cardiovascular disease. J. Am. Coll. Cardiol. 68 (25), 2850–2870. 10.1016/j.jacc.2016.09.972 28007146

[B150] Veech R. L. Chance B. Kashiwaya Y. Lardy H. A. Cahill G. F. Jr. (2001). Ketone bodies, potential therapeutic uses. IUBMB Life 51 (4), 241–247. 10.1080/152165401753311780 11569918

[B151] von Haehling S. Anker S. D. Bassenge E. (2003). Statins and the role of nitric oxide in chronic heart failure. Heart Fail Rev. 8 (1), 99–106. 10.1023/a:1022103222857 12652163

[B152] Voros G. Ector J. Garweg C. Droogne W. Van Cleemput J. Peersman N. (2018). Increased cardiac uptake of ketone bodies and free fatty acids in human heart failure and hypertrophic left ventricular remodeling. Circ. Heart Fail 11 (12), e004953. 10.1161/CIRCHEARTFAILURE.118.004953 30562098

[B153] Wallenius K. Kroon T. Hagstedt T. Lofgren L. Sorhede-Winzell M. Boucher J. (2022). The SGLT2 inhibitor dapagliflozin promotes systemic FFA mobilization, enhances hepatic β-oxidation, and induces ketosis. J. Lipid Res. 63 (3), 100176. 10.1016/j.jlr.2022.100176 35120993 PMC8953658

[B154] Wallner M. Eaton D. M. Berretta R. M. Liesinger L. Schittmayer M. Gindlhuber J. (2020). HDAC inhibition improves cardiopulmonary function in a feline model of diastolic dysfunction. Sci. Transl. Med. 12 (525), eaay7205. 10.1126/scitranslmed.aay7205 31915304 PMC7065257

[B155] Wang P. Tate J. M. Lloyd S. G. (2008). Low carbohydrate diet decreases myocardial insulin signaling and increases susceptibility to myocardial ischemia. Life Sci. 83 (25-26), 836–844. 10.1016/j.lfs.2008.09.024 18951908 PMC2642968

[B156] Weis E. M. Puchalska P. Nelson A. B. Taylor J. Moll I. Hasan S. S. (2022). Ketone body oxidation increases cardiac endothelial cell proliferation. EMBO Mol. Med. 14 (4), e14753. 10.15252/emmm.202114753 35179309 PMC8988203

[B157] Williams-Karnesky R. L. Sandau U. S. Lusardi T. A. Lytle N. K. Farrell J. M. Pritchard E. M. (2013). Epigenetic changes induced by adenosine augmentation therapy prevent epileptogenesis. J. Clin. Investig. 123 (8), 3552–3563. 10.1172/JCI65636 23863710 PMC3726154

[B158] Writing Committee M. Members A. A. J. C. (2022). 2022 AHA/ACC/HFSA guideline for the management of heart failure. J. Card. Fail 28 (5), e1–e167. 10.1016/j.cardfail.2022.02.010 35378257

[B159] Wu P. Vaseghi M. (2020). The autonomic nervous system and ventricular arrhythmias in myocardial infarction and heart failure. Pacing Clin. Electrophysiol. 43 (2), 172–180. 10.1111/pace.13856 31823401 PMC7723010

[B160] Xie Z. Zhang D. Chung D. Tang Z. Huang H. Dai L. (2016). Metabolic regulation of gene expression by histone lysine β-hydroxybutyrylation. Mol. Cell. 62 (2), 194–206. 10.1016/j.molcel.2016.03.036 27105115 PMC5540445

[B161] Yamada K. Takizawa S. Ohgaku Y. Asami T. Furuya K. Yamamoto K. (2020). MicroRNA 16-5p is upregulated in calorie-restricted mice and modulates inflammatory cytokines of macrophages. Gene 725, 144191. 10.1016/j.gene.2019.144191 31654705

[B162] Yang M. Zhang Y. Ren J. (2020). Acetylation in cardiovascular diseases: molecular mechanisms and clinical implications. Biochim. Biophys. Acta Mol. Basis Dis. 1866 (10), 165836. 10.1016/j.bbadis.2020.165836 32413386

[B163] Youm Y. H. Nguyen K. Y. Grant R. W. Goldberg E. L. Bodogai M. Kim D. (2015). The ketone metabolite β-hydroxybutyrate blocks NLRP3 inflammasome-mediated inflammatory disease. Nat. Med. 21 (3), 263–269. 10.1038/nm.3804 25686106 PMC4352123

[B164] Yurista S. R. Chong C. R. Badimon J. J. Kelly D. P. de Boer R. A. Westenbrink B. D. (2021b). Therapeutic potential of ketone bodies for patients with cardiovascular disease: JACC state-of-the-art review. J. Am. Coll. Cardiol. 77 (13), 1660–1669. 10.1016/j.jacc.2020.12.065 33637354

[B165] Yurista S. R. Matsuura T. R. Sillje H. H. W. Nijholt K. T. McDaid K. S. Shewale S. V. (2021a). Ketone ester treatment improves cardiac function and reduces pathologic remodeling in preclinical models of heart failure. Circ. Heart Fail 14 (1), e007684. 10.1161/CIRCHEARTFAILURE.120.007684 33356362 PMC7819534

[B166] Yurista S. R. Sillje H. H. W. Oberdorf-Maass S. U. Schouten E. M. Pavez Giani M. G. Hillebrands J. L. (2019). Sodium-glucose co-transporter 2 inhibition with empagliflozin improves cardiac function in non-diabetic rats with left ventricular dysfunction after myocardial infarction. Eur. J. Heart Fail 21 (7), 862–873. 10.1002/ejhf.1473 31033127

[B167] Zambrano A. K. Cadena-Ullauri S. Guevara-Ramirez P. Frias-Toral E. Ruiz-Pozo V. A. Paz-Cruz E. (2023). The impact of a very-low-calorie ketogenic diet in the gut microbiota composition in obesity. Nutrients 15 (12), 2728. 10.3390/nu15122728 37375632 PMC10305724

[B168] Zannad F. Ferreira J. P. Pocock S. J. Anker S. D. Butler J. Filippatos G. (2020). SGLT2 inhibitors in patients with heart failure with reduced ejection fraction: a meta-analysis of the EMPEROR-Reduced and DAPA-HF trials. Lancet 396 (10254), 819–829. 10.1016/S0140-6736(20)31824-9 32877652

[B169] Zhang D. Yang H. Kong X. Wang K. Mao X. Yan X. (2011). Proteomics analysis reveals diabetic kidney as a ketogenic organ in type 2 diabetes. Am. J. Physiol. Endocrinol. Metab. 300 (2), E287–E295. 10.1152/ajpendo.00308.2010 20959534

[B170] Zhang S. J. Li Z. H. Zhang Y. D. Chen J. Li Y. Wu F. Q. (2021a). Ketone body 3-hydroxybutyrate ameliorates atherosclerosis via receptor gpr109a-mediated calcium influx. Adv. Sci. (Weinh) 8 (9), 2003410. 10.1002/advs.202003410 33977048 PMC8097358

[B171] Zhang W. Guo X. Chen L. Chen T. Yu J. Wu C. (2021b). Ketogenic diets and cardio-metabolic diseases. Front. Endocrinol. (Lausanne) 12, 753039. 10.3389/fendo.2021.753039 34795641 PMC8594484

[B172] Zhang Y. Zhou S. Zhou Y. Yu L. Zhang L. Wang Y. (2018). Altered gut microbiome composition in children with refractory epilepsy after ketogenic diet. Epilepsy Res. 145, 163–168. 10.1016/j.eplepsyres.2018.06.015 30007242

[B173] Zhong Z. Huang M. Lv M. He Y. Duan C. Zhang L. (2017). Circular RNA MYLK as a competing endogenous RNA promotes bladder cancer progression through modulating VEGFA/VEGFR2 signaling pathway. Cancer Lett. 403, 305–317. 10.1016/j.canlet.2017.06.027 28687357

[B174] Ziegler A. Zaugg C. E. Buser P. T. Seelig J. Kunnecke B. (2002). Non-invasive measurements of myocardial carbon metabolism using *in vivo* 13C NMR spectroscopy. NMR Biomed. 15 (3), 222–234. 10.1002/nbm.764 11968138

[B175] Zordoky B. N. Sung M. M. Ezekowitz J. Mandal R. Han B. Bjorndahl T. C. (2015). Metabolomic fingerprint of heart failure with preserved ejection fraction. PLoS One 10 (5), e0124844. 10.1371/journal.pone.0124844 26010610 PMC4444296

[B176] Zou Z. Sasaguri S. Rajesh K. G. Suzuki R. (2002). dl-3-Hydroxybutyrate administration prevents myocardial damage after coronary occlusion in rat hearts. Am. J. Physiol. Heart Circ. Physiol. 283 (5), H1968–H1974. 10.1152/ajpheart.00250.2002 12384475

