# Supplementary material for: Underlying mechanisms of ketotherapy in heart failure: current evidence for clinical implementations
Source: Front Pharmacol. 2024 Oct 24;15:1463381. doi: 10.3389/fphar.2024.1463381 (PMC11540999; doi:10.3389/fphar.2024.1463381)
Supplement: Supplementary file 1 [file Table1.DOCX]

# Supplementary Materials

# Underlying Mechanisms of Ketotherapy in Heart Failure: current evidence for clinical implementations

Kun Liu^1^, Shao-chi Wang^1^, Hua-hu Guo^2^

^1^ Clinical Systems Biology Laboratories, the First Affiliated Hospital, Academy of Medical Sciences, Zhengzhou University, Zhengzhou, China.

^2^ Department of surgery, the First Affiliated Hospital, Zhengzhou University, Zhengzhou, China.

Correspondence to Hua-hu Guo, guohuahusci@163.com.

**Supplemental Tables:**

Table S1. Clinical Trials Utilizing Ketosis for Heart Failure

**Table S1. Clinical Trials Utilizing Ketosis for Heart Failure**

| **Intervention** | **Authors,**  **Year** | **TrialID** | **Intervention** | **Condition** | **Major outcomes** |
| --- | --- | --- | --- | --- | --- |
| Ketogenic Diets | Darlene et al., 2019 | NCT04942548 | Ketogenic Diets | N=25, HF | Diet-induced ketosis improved right ventricular function, suppressed NLRP3 inflammasome activation, and suppressed right ventricular fibrosis. |
|  | Adrian et al., 2023 | NCT04921293 | Ketogenic Diets | N=18, HFrEF & Ketosis | Awaiting study outcome. (Study the effect of ketogenic diets on symptoms in clinical severity,liquid retention,ejection fraction improvement.) |
|  | Mahavir et al., 2023 | NCT05571865 | Probiotic | N=40, Diabetic Cardiomyopathies;HF | Awaiting study outcome. (Study the effect of probiotic on glucose in blood and urine and Biochemical indexes) |
|  | Yuchi Han et al., 2023 | NCT06081543 | Ketogenic Diets | N=90, HFrEF | Awaiting study outcome. (Study the effect of ketogenic diets on exercise and cardiac output) |
| Ketone Infusion | Roni et al., 2017 | NCT03073356 | β-HB infusion | N=44, HF | β-HB increases MVO2 without changing MEE and benefits hemodynamic effects in patients with HFrEF without affecting MEE. |
|  | Nigopan et al., 2021 | NCT04703361 | β-HB infusion | N=12, HF & Ketonemia | β-HB acutely increased CO and affected hemodynamics, which was not altered by aspirin and niacin. |
|  | Ralph et al., 2019 | NCT03560323 | β-HB infusion | N=78, HF & Type 2 Diabetes | Awaiting study outcome. (Study the effect of β-HB on cardiac function, myocardial glucose uptake and energetics) |
| Oral  Ketones | J. Eduardo et al., 2020 | NCT04370600 | β-HB ester | N=16, Chronic HF | Unknown status. (Study the effect of β-HB ester on cardiac output) |
|  | Hans et al., 2020 | NCT04379934 | β-HB | N=40, HF & Mitochondrial Pathology | Unknown status. (Study the effect of β-HB on contractile force, mitochondrial respiratory capacity and reactive oxygen production) |
|  | Kristian et al., 2020 | NCT04442555 | Ketone Ester | N=12, Acute HF | Awaiting study outcome. (Study the effect of β-HB ester on cardiac output) |
|  | Kristian et al., 2019 | NCT04443426 | 2-hydroxybutyrate salts\|Ketone Monoester | N=8, HF & Ketosis | Awaiting study outcome. (Study the effect of β-HB ester on cardiac output) |
|  | Kristian et al., 2020 | NCT04594265 | KetoneAid KE4 Pro Monoester | N=8, HF & Ketosis | Awaiting study outcome. (Study the effect of β-HB ester on cardiac output) |
|  | Kenneth et al., 2020 | NCT04633460 | Ketone ester | N=20, HFrEF | Not reported. (Study the effect of β-HB ester on systemic vascular resistance, substrate utilization, VO2 efficiency,exercise-induced arrhythmias) |
|  | Marek et al., 2020 | NCT04698005 | Ketone monoester | N=24, HF & Ketosis | Unknown status. (Study the effect of β-HB ester on symptoms in patients with acute Heart Failure) |
|  | Kristoffer et al., 2022 | NCT05161650 | Ketone monoester | N=26, HF | Not reported. (Study the effect of ketone monoester on cardiopulmonary function) |
|  | Kristoffer et al., 2021 | NCT05161676 | Ketone monoester | N=12, HF | Not reported. (Study the effect of ketone monoester on lipolysis, protein metabolism, glucose kinetic) |
|  | Kristoffer et al., 2022 | NCT05236335 | Ketone ester | N=24, HFrEF & Type 2 Diabetes | Not reported. (Study the effect of ketone ester on cardiopulmonary function) |
|  | Daan et al., 2022 | NCT05348460 | Ketone ester | N=20, HFrEF | Awaiting study outcome. (Study the effect of Ketone ester on exercise and metabolism) |
|  | Université de Sherbrooke, 2022 | NCT05651529 | Ketone salt | N=20, HF | Awaiting study outcome. (Study the effect of Ketone salt on cardiac output, glucose in blood and urine and Biochemical  indexes) |
|  | Senthil et al., 2023 | NCT05757193 | Ketone ester | N=20, HFrEF | Not reported. (Study the effect of ketone ester on cardiac output) |
|  | University of Aarhus, 2023 | NCT05768100 | 1,3-Butanediol | N=12, HF | Awaiting study outcome. (Study the effect of 1,3-Butanediol on cardiac output) |
|  | Christopher et al., 2023 | NCT05924802 | Ketone Ester | N=50, HF | Awaiting study outcome. (Study the effect of ketone ester on cardiac output) |
|  | Yuchi Han et al., 2023 | NCT06078683 | Ketone ester | N=30, HFrEF & Type 2 Diabetes | Awaiting study outcome. (Study the effect of Ketone ester on exercise and cardiac output) |
|  | Carolina et al., 2023 | NCT06108076 | Ketone Monoester (KE) | N=10, HFrEF & Type 2 Diabetes | Awaiting study outcome. (Study the effect of acute Ketone ester on cardiac output and metabolism) |
|  | Senthil et al., 2023 | NCT06195982 | ketone ester | N=25, HFrEF | Awaiting study outcome. (Study the effect of Ketone ester on cardiac output and exercise capacity) |
| SGLT2i | Jesper et al., 2017 | NCT03198585 | Empagliflozin | N=190, HFrEF | Empagliflozin increased plasma GDF-15 levels but not hsTNT and hsCRP in patients with HFrEF. |
|  | mkort et al.,2018 | NCT03554200 | Empagliflozin | N=19, HF & Type 2 Diabetes | Terminated. (Study the effect of empagliflozin on cardiac output, hemodynamics,systemic quality of life) |
|  | Trevor et al., 2020 | NCT04071626 | Ertugliflozin | N=9, HF & Type 2 Diabetes | Terminated. (Study the effect of ertugliflozin on Peak VO2, Left Ventricular Mass Index) |
|  | Morten et al., 2021 | NCT05042973 | Empagliflozin | N=120, HF & Obesity | Awaiting study outcome. (Study the effect of empagliflozin on symptoms and cardiac function) |
|  | Carolina et al., 2021 | NCT05057806 | Empagliflozin | N=30, HFrEF & Type 2 Diabetes | Awaiting study outcome. (Study the effect of empagliflozin on cardiopulmonary function) |
|  | Payman et al., 2022 | NCT05138575 | Empagliflozin | N=53, HFrEF | Awaiting study outcome. (Study the effect of empagliflozin on cardiopulmonary function and submaximal exercise endurance) |
|  | Eli Lilly and Company, 2022 | NCT05262764 | JARDIANCE | N=1201, HF | Awaiting study outcome. (Study the effect of JARDIANCE on the incidence of adverse drug reactions, all-cause death, cardiovascular (CV) death) |
|  | Kenneth et al., 2023 | NCT05885607 | - | N=40, HF | Awaiting study outcome. (Study the effect of SGLT2i on systemic metabolomic and proteomic profiles) |
|  | Maastricht University Medical Center, 2023 | NCT06046612 | Empagliflozin | N=48, HFrEF & Microvascular Dysfunction | Awaiting study outcome. (Study the effect of empagliflozin on cutaneous vascular conductance and outcome) |
|  | Alessandro et al., 2024 | NCT06217302 | Sotagliflozin | N=150, HF & Type 1 Diabetes | Awaiting study outcome. (Study the effect of sotagliflozin on kidney function) |
|  | Ralph et al., 2024 | NCT06229678 | Empagliflozin | N=71, HFrEF & Type 2 Diabetes | Awaiting study outcome. (Study the effect of empagliflozin on cardiac output and myocardial blood flow) |
| DRUG: Semaglutide | Barry et al., 2022 | NCT05371496 | Semaglutide | N=81, HFpEF & Obesity | Awaiting study outcome. (Study the effect of semaglutide on cardiopulmonary function) |

* Data were collected on international Clinical Trials registry platform (ICTRP, https://trialsearch.who.int/)
